# Adverse events associated with the use of cannabis-based products in people living with cancer: a systematic scoping review

**DOI:** 10.1007/s00520-024-09087-w

**Published:** 2024-12-18

**Authors:** Irene Cheah, Jennifer Hunter, Ingrid Gelissen, Wai-Jo Jocelin Chan, Joanna E. Harnett

**Affiliations:** 1 School of Pharmacy, Faculty of Medicine and Health, University of Sydney, Sydney, Australia; 2 Health Research Group, Sydney, Australia

**Keywords:** Adverse events, Cannabis, Randomised controlled trial

## Abstract

**Purpose:**

To summarise the extent and type of evidence in relation to adverse events (AEs) associated with the use of cannabis-based products (CBP) in people living with cancer.

**Methods:**

The Joanna Briggs Institute (JBI) methodology for scoping reviews was applied. A search was performed in MEDLINE (Ovid), Embase (Ovid), CINAHL (EBSCOhost), Scopus, Web of Science Core Collections and AMED (Ovid) from their inception to 7 May 2023. Primary studies reporting AEs associated with any form of natural or synthetic CBP use in any cancer care setting and location were included.

**Results:**

One hundred fifty-two studies were included, with the most prevalent being randomised controlled trials (RCTs) (*n* = 61), followed by non-randomised controlled trials (*n* = 26) and case reports (*n* = 23). CBP was mainly used in gastrointestinal, liver, or peritoneal cancer (*n* = 98) and haematological or lymphoid cancer (*n* = 92), primarily to manage nausea and vomiting (*n* = 78) and cancer pain (*n* = 37). The most common CBP ingredients were combinations of THC and CBD (*n* = 69), synthetic THC (*n* = 47), single compounds of THC (*n* = 42) and CBD (*n* = 16) with diverse forms, administration routes and doses. The primary methods of administration were oral (*n* = 94) and inhalation (*n* = 54). A broad range of AEs were reported; the most common were related to the nervous system (*n* = 118), psychiatric (*n* = 101) and gastrointestinal system (*n* = 81). Diverse patient characteristics, significant under-reporting and low-quality reporting were observed in many studies.

**Conclusions:**

More rigorous research designs that prioritise comprehensive, standardised reporting of AEs and CBP use are required to fully elucidate the safety profile of CBP use in cancer care.

**Supplementary Information:**

The online version contains supplementary material available at 10.1007/s00520-024-09087-w.

## Introduction

Cannabis-based products (CBP) are used by people living with cancer to manage cancer symptoms and side effects of conventional therapy, improve quality of life and promote general health [1]. The prevalence of CBP use in this population is estimated to be 20–48%, with increased use reported in the last 10 years [2–11].

Cannabis is a flowering plant belonging to the Cannabaceae family. *Cannabis sativa*, *Cannabis indica*, *Cannabis ruderalis* and hybrids of these species are the most commonly used for medicinal purposes*.* The plant contains a broad range of chemical compounds including cannabinoids, terpenes and flavonoids, which are attributed to therapeutic benefits and side effects [12].

Key constituents in CBP, namely cannabinoids, mimic the effects of endogenous cannabinoids by binding to CB1 and CB2 receptors distributed throughout the body and activating the cannabinoid system. CB1 is found predominantly in the central nervous system and CB2 in immune cells. Two notable cannabinoids found in cannabis are delta 9-tetrahydrocannabinol (Δ9-THC) and cannabidiol (CBD). Δ9-THC is the main psychoactive component causing euphoria, relaxation, anxiety or hallucinations. CBD is non-psychoactive and studied for its anti-inflammatory, analgesic, anxiolytic and neuroprotective properties [1, 12]. In addition, various synthetic analogues are available.

CBP-containing cannabinoids are available in various forms. The dried parts of the plant (leaves, flowers, buds), extracts (oil, tincture), edibles (food, beverages), topical applications (creams, gels, oil) and synthetic forms (capsules, tablets, solutions, suppositories) are administered by inhalation (by smoking or vaporising), oral ingestion, sublingual, topical or rectal application [13].

Access to CBP is influenced by the legal frameworks of a given country or jurisdiction. An increasing number of jurisdictions are now allowing legal access to medicinal cannabis for use in cancer care [14, 15]. This reflects shifting societal attitudes and healthcare approaches and an emerging evidence-base to support its use in cancer care [16–19]. The process of such legalisation requires the establishment of regulatory frameworks that oversee the production, sale and consumption of CBP.

Clinical concerns about the safety of CBP use by people living with cancer are warranted as they are potentially a population at increased risk of adverse effects. Knowledge about adverse events (AEs) is crucial for making informed decisions and delivering safe, evidence-based care.

To date, no review has comprehensively reported on AEs associated with the use of any type of CBP in the cancer care context. Therefore, the aim of this review was to summarise the literature reporting AEs associated with CBP use by people living with cancer.

## Methods

The Joanna Briggs Institute (JBI) methodology for scoping reviews [20] was employed for this study, and reported according to the Preferred Reporting Items for Systematic Reviews and Meta-Analyses Extension for Scoping Reviews (PRISMA-ScR) [21] and relevant items from the updated PRISMA 2020 statement [22] (S1). The review protocol was submitted for publication prior to study screening and selection [23].

### Search strategy

The search strategy was developed for MEDLINE (Ovid) (S2) in consultation with an academic librarian and adapted for Embase (Ovid), CINAHL (EBSCOhost), Scopus, Web of Science Core Collections and AMED (Ovid). Literature published from inception to 7 May 2023, with no language restrictions, was searched using three concepts and related search terms: cancer diagnoses, adverse effects and CBP (S3). Due to the breadth of papers identified, no hand searches were conducted for additional articles.

### Eligibility criteria

The JBI framework for scoping review eligibility criteria of Population, Concept and Context (PCC) was applied [20].

### Population

Included were people of any age, gender or ethnicity, living with any cancer type and stage and comorbidities and using CBPs with or without concurrent treatments or recreational use of tobacco/alcohol/drugs. Mixed populations, including palliative care where only some participants have cancer, were only included when data was reported for the cancer population subgroup. Studies reporting or evaluating the risk of developing cancer associated with CBP use in other populations were excluded.

### Concept

The concept of interest was AEs associated with any form of CBP use in cancer. Here, CBP refers to all forms of cannabis including natural and synthetic products and recreational and medicinal products (registered or unregistered).

We adopted The International Conference on Harmonisation of Technical Requirements for Registration of Pharmaceuticals for Human Use definition of an AE as being “any untoward medical occurrence in a patient administered a medicinal product and which does not necessarily have to have a causal relationship with this treatment. An AE can therefore be any unfavourable and unintended sign (for example, an abnormal laboratory finding), symptom, or disease temporally associated with the use of a medicinal product, whether or not considered related to this medicinal product” and a serious AE as “any untoward medical occurrence that at any dose results in death, is life-threatening, requires inpatient hospitalisation or prolongation of existing hospitalisation, results in persistent or significant disability/incapacity, is a congenital anomaly/birth defect, or is a medically important event or reaction” [24].

### Context

AEs that occurred in any setting such as home, primary care, secondary care or palliative care across any geographical location were included. There were no limitations on the individuals identifying or reporting the AE, nor on the reasons for using CBP within the cancer setting, including recreational use.

### Types of studies

Primary data sources including randomised controlled trials, non-randomised controlled trials, observational studies, case–control studies, case series, case reports, cross-sectional surveys and retrospective chart reviews were included. Preclinical studies and secondary data sources such as systematic reviews, meta-analyses and clinical guidelines were excluded. Only articles published in peer review journals were included. Non-English articles; grey literature such as newspaper reports, white papers and university theses; and data from adverse events reporting systems or clinical trial databases were excluded. A post hoc decision was made to exclude conference meeting abstracts reporting the findings of potentially eligible studies or case studies with very low-quality non-specific AE reporting.

### Source of evidence selection

All citations retrieved in the search were managed using Covidence [25] for automatic and manual identification of duplicate records, screening and selecting studies and data extraction.

A calibration exercise was conducted by two authors using the first 100 articles, to refine the full-text exclusion categories. Titles, abstracts and full-text articles were independently screened by two reviewers. Any disagreements that arose during the screening and selection processes were resolved through discussions with the other reviewers.

### Data extraction and analysis

After piloting and refining the data extraction template in Covidence, data were recorded by a single reviewer and verified by another. The study design, country/region, participant characteristics, reason for CBP use, CBP intervention, use of a comparator, concomitant interventions, recreational tobacco/alcohol/drug use, AE characteristics and author’s conclusion or comments were recorded. Authors of the included studies were not contacted to request for missing or additional data. The data extraction template is provided in S4.

To optimise clarity and consistency in reporting across the included studies, each AE was then categorised according to the Common Terminology Criteria (CTCAE) System Organ Class (SOC) [26]. Overarching categories were also created to summarise the different ingredients and administration routes of the CBP. Due to high heterogeneity in the data, categories for dose, duration and other exposures were not created and not reported using descriptive statistics. One author re-coded this data and a second verified the coding.

During data extraction and coding, any disagreements were resolved through discussions with the other reviewers. As the aim was to map and characterise the available evidence, a critical appraisal and risk of bias assessments were not conducted, as per scoping review methodology [20]. Microsoft Excel [27] was used to generate charts and figures. Other findings were summarised and narrated.

## Results

### Study selection

**Fig. 1:**
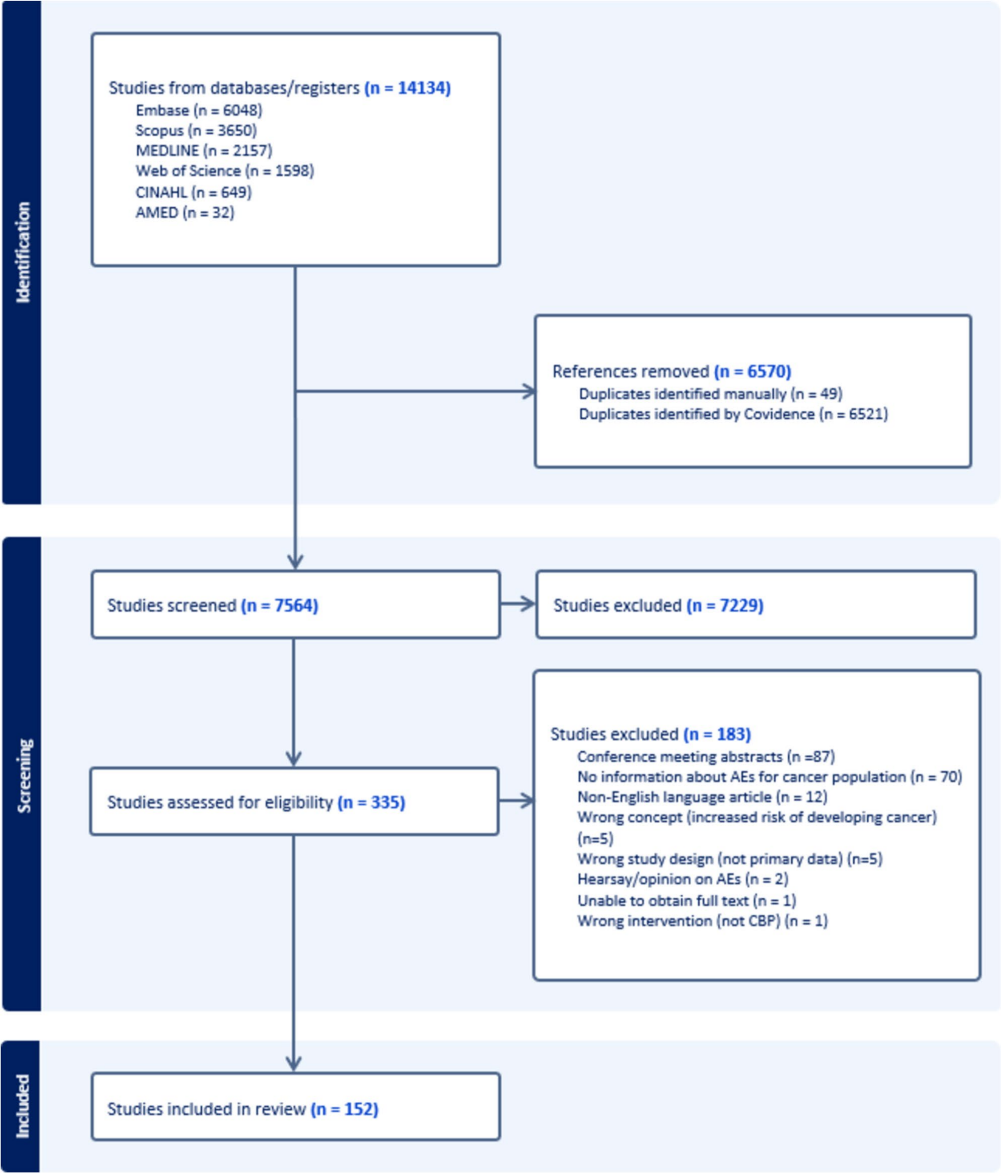
Flow chart of database search and study selection

An initial 14,134 records were identified across the databases searched. Following the removal of duplicate articles (*n* = 6570) and those not meeting the inclusion criteria for title and abstract (*n* = 7229), 335 full-text articles were subject to full-text review. Of these, an additional 183 studies were excluded. These are reported in S5 along with the reasons for exclusion. A total of 152 articles were included [28–179]. Figure 1 presents the study selection process by stages [22].

### Study characteristics

Table 1 summarises key characteristics of the 152 included studies which were published between 1974 and 2023. Study designs included randomised controlled trials (RCTs) (*n* = 61), non-randomised controlled trials (*n* = 26), case reports (*n* = 23) and cross-sectional surveys (*n* = 17). Most studies (72.4%) included participants from both sexes. Information about ethnicity was under-reported in most studies.

**Table 1:** Study characteristics (*n* = 152)

Study design	No. (%)	Citations
RCT	57 (37.5%)	[29, 40, 44, 45, 47, 49–51, 53, 56, 57, 61–63, 65, 67, 70–72, 74, 76, 79, 83, 85–88, 93, 95, 98, 99, 101, 103, 113, 114, 118, 120, 123–126, 128, 133, 134, 144, 145, 147, 150, 152–155, 165, 166, 168, 169, 179]
Non-randomised controlled trial	24 (15.8%)	[30, 48, 52, 58–60, 68, 69, 75, 78, 80, 89, 90, 100, 104, 111, 116, 119, 121, 156, 159, 162, 167, 175]
Case report	23 (15.1%)	[42, 55, 81, 82, 84, 94, 96, 108–110, 115, 117, 129, 135–137, 142, 149, 151, 158, 160, 164, 170]
Cross-sectional survey	17 (11.2%)	[46, 73, 77, 107, 122, 130, 131, 140, 143, 146, 148, 163, 171, 174, 176–178]
Prospective single-arm observational studies	9 (5.9%)	[28, 31–35, 38, 39, 127]
Retrospective chart review	8 (5.3%)	[64, 66, 97, 112, 132, 139, 157, 161]
Case series	8 (5.3%)	[41, 54, 91, 92, 102, 138, 172, 173]
Case–control study	2 (1.3%)	[36, 37]
RCT with observational extension	2 (1.3%)	[43, 106]
RCT and non-randomised controlled trial	2 (1.3%)	[105, 141]
**Country**	No. (%)*	**Citations**
USA	69 (45.4%)	[30, 31, 41, 44, 45, 47, 52, 54, 58–60, 62, 64–68, 70, 74, 79, 80, 84, 88, 90, 92, 93, 98–100, 103, 104, 106–108, 113, 114, 117–119, 125, 126, 128, 130, 131, 134–136, 139–141, 143–145, 150, 152, 154, 157–159, 163, 164, 167–169, 172–174, 177, 179]
Canada	19 (12.5%)	[32, 40, 42, 43, 46, 50, 77, 85, 101, 102, 109–112, 129, 132, 149, 176]
UK	18 (11.8%)	[29, 51, 53, 56, 57, 65, 83, 86, 87, 91, 103, 105, 133, 138, 156, 166, 175]
Israel	16 (10.5%)	[28, 33–39, 65, 96, 127, 146, 160, 161, 171, 178]
Germany	9 (5.9%)	[65, 69, 78, 103, 120, 148, 155, 166]
Australia	9 (5.9%)	[48, 63, 65, 71, 72, 76, 81, 147, 151]
Other^†^	55 (36.2%)	[49, 55, 61, 65, 73, 75, 82, 87, 89, 94, 95, 97, 103, 115, 116, 121–124, 134, 137, 142, 153, 155, 162, 165, 166, 170]
**Participant age group**	**No. (%)**^‡^	**Citations**
Adult (18–64 years)	132 (86.8%)	[29–41, 44–49, 52–55, 57–73, 75–93, 95, 96, 98–101, 103–107, 110–114, 116–124, 127–136, 138–148, 150, 153–174, 176–179]
Elderly (65 years and above)	87 (57.2%)	[29, 31, 32, 42, 52, 53, 57, 60–62, 64–68, 72, 73, 75–80, 83, 85–91, 99–101, 103–107, 109, 111–116, 119, 121–124, 128, 130, 131, 133, 134, 139–141, 143–145, 147–150, 153–157, 159, 161–163, 165–169, 171, 172, 174, 176–179]
Child (1–17 years)	28 (18.4%)	[28, 31, 35, 43–46, 52, 56, 58, 62, 63, 79, 83, 91, 94, 100, 102, 108, 127, 132, 137, 141, 144, 148, 151, 173, 176]
Infant (below 1 year)	5 (3.3%)	[31, 46, 56, 127, 148]
Not defined	11 (7.2%)	[33, 34, 50, 51, 74, 77, 97, 125, 126, 152, 175]
**Cancer diagnosis**	**No. (%)**^§^	**Citations**
Gastrointestinal/liver/peritoneal	98 (64.5%)	[32–35, 37–42, 48, 54, 67, 68, 71–73, 76, 77, 80, 85, 87, 97–99, 107, 109, 112–114, 119, 122, 125, 126, 134, 138, 139, 155, 157, 159, 162, 163, 167, 171, 176–179]
Haematological/lymphoid	92 (60.5%)	[28, 32, 33, 35, 38, 41, 46, 48, 54, 56, 59, 62, 69, 71–73, 76, 77, 80, 82, 88, 89, 92, 95, 98, 99, 102, 104, 106, 107, 109, 112–114, 116, 117, 119, 122, 125–127, 132, 133, 138, 139, 141, 143, 146, 150, 151, 155, 159, 160, 163, 164, 168, 176–179]
Lung/thoracic	59 (38.8%)	[29, 32–40, 48, 57, 59, 61, 69, 72, 73, 76–78, 80, 85, 86, 88, 89, 97–99, 101, 104, 106, 107, 112, 114, 119, 122–125, 133, 134, 139, 141, 149, 150, 155, 157–159, 161–163, 165, 167, 170, 171, 176, 178, 179]
Breast	53 (34.9%)	[32–35, 37, 38, 40, 41, 48, 53, 54, 59, 61, 69–72, 76–78, 84, 86–88, 91, 97–99, 101, 104–107, 112, 113, 119, 122, 125, 126, 129, 133, 134, 139, 141, 150, 157, 159, 162, 163, 171, 176, 177, 179]
Urogenital	45 (29.6%)	[32, 33, 35, 36, 39, 40, 48, 53, 54, 62, 69, 71, 72, 76, 77, 80, 86–89, 97, 99, 104, 106, 112–114, 119, 120, 122, 125, 127, 133, 134, 138, 139, 142, 155, 157, 161, 163, 176–179]
Gynaecological	43 (28.3%)	[32–35, 48, 51, 53, 54, 61, 66, 71, 72, 76, 77, 88, 89, 97, 101, 104, 106, 112–115, 122, 125, 126, 133, 138, 139, 141, 150, 155, 157, 159, 162, 163, 167, 171, 174, 176–178]
Other (location not specified)^¶^	38 (25%)	[32–34, 36–38, 46, 53, 58, 59, 72, 76, 77, 79, 85, 88, 96, 107, 114, 119, 125, 127, 128, 132, 134, 139, 141, 143, 155, 157, 162, 163, 167, 168, 171, 176]
Sarcoma	34 (22.4%)	[32, 33, 39, 44, 45] [46, 56, 62, 69, 71, 77, 78, 80, 89, 104, 113, 125, 127, 133] [87, 122, 138, 139, 150, 159, 162, 168, 176] [162]
Neurological	33 (21.7%)	[30, 32, 33, 35, 38, 46, 48, 55, 56, 75, 77, 80, 81, 90, 91, 94, 102, 104, 112, 122, 127, 132, 137, 140, 147, 157, 162, 163, 166, 173, 176, 177]
Head/neck	22 (14.5%)	[32, 33, 39, 48, 50, 56, 64, 77, 98, 104, 110, 112, 122, 125, 135, 136, 139, 155, 157, 162, 163]
Skin	20 (13.2%)	[33, 35, 36, 39, 48, 70, 77, 78, 80, 88, 89, 96, 104, 113, 122, 125, 133, 161, 162, 176]
Endocrine/thyroid	9 (5.9%)	[33, 51, 73, 97, 108] [119, 125, 163, 167]
Not reported	36 (23.7%)	[31, 33, 43, 46, 47, 49, 52, 60, 63, 65, 71, 74, 77, 83, 93, 100, 101, 103, 105, 111, 118, 119, 121, 125, 130, 131, 134, 144, 145, 148, 152–154, 156, 169, 172, 175]
**Cancer stage**	**No. (%)**	**Citations**
Advanced	47 (30.9%)	[36, 37, 39–42, 48, 55, 65, 71, 74–76, 78, 81, 84–87, 90, 91, 96, 103, 105, 108–111, 117, 119, 125, 133–137, 147, 149, 151, 153, 155, 157, 161, 165, 166, 170, 179]
Mixed	24 (15.8%)	[29, 33, 34, 50, 54, 61, 66–69, 72, 73, 104, 107, 116, 139, 143, 146, 163, 171, 173, 174, 176, 177]
Early stage	3 (2%)	[82, 99, 129]
Not reported	78 (51.3%)	[28, 30–32, 35, 38, 43–47, 49, 51–53, 56–60, 62–64, 70, 77, 79, 80, 83, 88, 89, 92–95, 97, 98, 100–102, 106, 112–115, 118, 120–124, 126–128, 130–132, 138, 140–142, 144, 145, 148, 150, 152, 154, 156, 158–160, 162, 164, 167–169, 172, 175, 178]
**Declaration**	**No. (%)**	**Citations**
Disclosed funding and interests	46 (30.3%)	[30–32, 34–36, 39, 48, 50, 54, 61, 65, 71, 72, 74, 76, 77, 84, 87, 90, 94, 96, 100, 103, 106, 108, 114, 116, 121, 129, 130, 139, 140, 143, 146, 149, 155, 157, 162, 163, 165, 166, 171, 173, 177, 179]
Disclosed either funding or interests	77 (50.7%)	[29, 33, 37, 40–47, 49, 51, 53, 56, 59, 63, 64, 66, 70, 73, 75, 78–83, 85, 86, 88, 89, 91–93, 95, 97–99, 101, 102, 104, 105, 111–113, 117–119, 122, 123, 125–128, 132, 134–138, 144, 145, 147, 148, 150, 154, 159, 161, 164, 167–169, 174–176, 178]
Did not disclose funding or interests	29 (19.1%)	[28, 38, 52, 55, 57, 58, 60, 62, 67–69, 107, 109, 110, 115, 120, 124, 131, 133, 141, 142, 151–153, 156, 158, 160, 170, 172]

*Six studies were conducted across multiple countries

^†^Switzerland, Romania, Belgium (*n* = 4); Spain, Hungary, Netherlands, Poland, Lithuania, Bulgaria (*n* = 3); Estonia, Finland, Czech Republic, Denmark, Latvia, India (*n* = 2); Sweden, Mexico, Taiwan, Europe, Austria, Italy, Hong Kong, Brazil, Thailand, France, South Africa, New Zealand, Latin America (*n* = 1)

^‡^Ninety-six studies included participants from multiple age groups

^§^One hundred four studies involved cancer diagnoses across multiple body systems

^¶^Included germ cell cancer (*n* = 2), adenocarcinoma (*n* = 2), solid tumours (*n* = 2), anaplastic carcinoma (*n* = 1), small cell cancer (*n* = 1), mesothelioma (*n* = 1)

CBP were used across a wide spectrum of cancer diagnoses, with gastrointestinal, liver or peritoneal cancer (64.5%) and haematological or lymphoid cancer (60.5%) being the most common. The most common exclusion criteria for participants across the included studies were psychiatric risk factors (50.0%), either a history of or current drug/alcohol/tobacco use (34.9%), cardiovascular risk factors (30.2%), hepatic risk factors (26.7%), renal risk factors (24.4%) and neurological risk factors (19.8%) risk factors.

Other characteristics of the included studies were the range of comparators and concomitant treatments being used. Depending on the reason for CBP use, the comparator interventions reported were placebo, prochlorperazine, thiethylperazine, levomepromazine, triflupromazine, metoclopramide, domperidone, dimenhydrinate, megestrol acetate, ondansetron, codeine and secobarbital. Reported concomitant treatments included biological therapy, chemotherapy, radiotherapy, immunotherapy, hormone therapy, opioids, other analgesics, corticosteroids, antiemetics, antiepileptics, psychotropics and cardiovascular therapy. However, a notable proportion of the studies (19.1%) did not report any information about concomitant interventions. Seventeen studies [46, 61, 64, 66, 77, 92, 131, 135, 136, 143, 146, 158, 160, 163, 168, 169, 174] reported participants’ recreational use of CBP.

### Cannabis-based products (CBP) characteristics and context

Table 2 summarises the characteristics of CBP (ingredient, form and route of administration) and the context of use.

**Table 2:** CBP characteristics and context of use (*n* = 152)

Ingredient	No. (%)*	Citations
Combined THC and CBD	69 (45.4%)	[31–35, 37–39, 48, 54, 61, 65, 66, 72, 74, 84, 86, 87, 91, 102, 103, 106, 110, 112, 116, 129, 130, 134, 135, 137, 140–143, 146, 147, 155, 157, 158, 160, 162, 163, 166, 177–179]
Synthetic THC	47 (30.9%)	[29, 30, 40, 41, 43, 50–53, 56, 59, 62, 66, 69, 70, 78–80, 83, 85, 88–90, 98–100, 105, 109, 111, 114, 120, 123, 124, 132, 133, 138, 148–150, 152–154, 156, 165, 167, 172, 175]
THC	42 (27.6%)	[28, 33, 35, 44, 45, 47, 49, 55, 57, 58, 60, 63, 67, 68, 71, 75, 86, 87, 93, 95, 96, 101, 104, 113, 118, 119, 125–128, 130, 136, 139, 141, 144, 145, 155, 159, 164, 168–170]
CBD	16 (10.5%)	[33, 54, 55, 71, 73, 76, 81, 91, 94, 108, 121, 127, 130, 139, 140, 173]
Hemp oil	1 (0.7%)	[151]
Not reported	20 (13.2%)	[32, 36, 42, 46, 64, 66, 77, 82, 92, 97, 107, 115, 117, 122, 130, 131, 161, 171, 174, 176]
**Route of administration**	**No. (%)**^†^	**Citations**
Oral	94 (61.8%)	[31–35, 37–39, 48, 54, 61, 65, 66, 72, 74, 84, 86, 87, 91, 102, 103, 106, 110, 112, 116, 129–131, 134, 135, 137, 140–143, 146, 147, 155, 157, 158, 160, 162, 163, 166, 177–179]
Inhaled	54 (35.5%)	[31–38, 42, 44–46, 55, 64, 66, 77, 107, 110, 117, 127, 131, 136, 139, 140, 143, 146, 157, 158, 160, 161, 163, 171, 174, 177–179]
Oromucosal	15 (9.9%)	[31, 48, 61, 65, 74, 77, 86, 87, 91, 103, 106, 116, 134, 157, 166]
Injection^‡^	11 (7.2%)	[52, 69, 75, 78, 83, 89, 90, 150, 152, 156, 167]
Topical	10 (6.6%)	[54, 77, 82, 110, 139, 143, 157, 174, 178, 179]
Sublingual	7 (4.6%)	[33, 34, 66, 96, 115, 139, 146, 174, 179]
Other^§^	5 (3.3%)	[27, 60, 62, 111, 133]
Rectal	2 (1.3%)	[77, 143]
Not reported	17 (11.2%)	[32, 66, 73, 81, 92, 97, 122, 130–132, 135, 138, 139, 148, 171, 173, 176]
**Context of use**	**No. (%)**^¶^	**Citations**
Nausea and vomiting	78 (51.3%)	[28–31, 34, 38, 42–45, 47, 49, 51–53, 55, 56, 58–63, 66–70, 72, 77–80, 83, 88, 89, 93, 95, 97–101, 104, 105, 107, 111–114, 118, 120, 122–124, 127, 128, 132, 133, 138–141, 144–146, 148, 150, 152, 154, 156, 159, 167–169, 171, 174, 175]
Cancer pain	37 (24.3%)	[31, 32, 34, 35, 48, 57, 65, 66, 74, 77, 86, 87, 103, 106, 107, 111, 112, 122, 125–127, 129, 130, 134, 137, 139, 140, 146, 148, 153, 162, 163, 170, 171, 174, 178, 179]
General/cancer care (non-specific symptoms)	25 (16.4%)	[33, 35–37, 46, 50, 57, 60, 64, 66, 71, 73, 76, 102, 107, 112, 122, 131, 143, 146, 147, 157, 158, 161, 163, 176]
Anorexia	21 (13.8%)	[31, 38, 39, 66, 77, 85, 97, 107, 111, 112, 119, 122, 139, 146, 148, 155, 165, 171, 172, 174, 178]
Other^#^	19 (12.5%)	[40, 41, 50, 66, 77, 96, 109, 110, 112, 122, 139, 146]
Mental health (anxiety, depression)	18 (11.8%)	[31, 38, 57, 66, 77, 97, 107, 112, 122, 127, 139, 140, 146, 148, 162, 163, 174, 178]
Insomnia	17 (11.2%)	[31, 35, 66, 77, 84, 97, 107, 112, 122, 127, 139, 140, 148, 163, 174, 177, 178]
Antitumoral action	16 (10.5%)	[75, 77, 82, 90, 91, 94, 108, 112, 116, 122, 139, 143, 151, 157, 166, 171]
Neuropathy	8 (5.3%)	[54, 112, 121, 129, 149, 164, 174, 178]
Fatigue	7 (4.6%)	[31, 35, 38, 66, 77, 96, 112]
Seizures	5 (3.3%)	[55, 81, 140, 148, 173]
Cachexia	3 (2%)	[39, 148, 155]
Weakness	2 (1.3%)	[35, 171]
Recreation	2 (1.3%)	[77, 136]
Not reported	6 (3.9%)	[92, 115, 117, 135, 142, 160]

*Thirteen studies involved multiple CBP ingredients

^†^Eighteen studies involved multiple routes of administration

^‡^Intramuscular *n* = 9, intravenous *n* = 1, intracranial *n* = 1

^§^Enteral *n* = 2, both ears *n* = 1, free form *n* = 1, intranasal *n* = 1

^¶^Thirty-three studies involved multiple CBP indications

^#^Appetite, arthralgia/myalgia, chemosensory perception, drowsiness, general discomfort, headache, itch, night sweats, other, palliative wound care, unclear purpose, quality of life, spasticity (vismodegib-related muscle cramps), strengthening body to combat cancer, toxicity, use as a supplement

#### CBP ingredient

The main CBP ingredients reported across the studies were combinations of THC and CBD (45.4%), followed by synthetic THC (30.9%) and single compounds of THC (27.6%) and CBD (10.5%), noting some studies reported use of more than one formulation. Sometimes, the exact ratio of THC to CBD was reported (e.g. 150:0, 15:0, 100:1, 20:1, 15:1, 2:1, 1:1, 1:2, 1:20, 1:50, 1:100), whilst other studies simply stated the CBP was balanced, high or low THC:CBD ratio, and for others, it was inferred (e.g. *Cannabis sativa* L., marijuana, mixed, dried cannabis, prescribed cannabis medication).

#### CBP form

A diverse range of forms of CBP were utilised across studies including capsules, oils, solutions, extracts, cigarettes, pastes, creams, ointments, sprays, infusions, lotions, suppositories, edibles, inflorescence, flowers and plants. There were substantial variations in the dosage and duration of administration.

#### CBP administration route

The administration routes of CBP varied widely. The predominant methods of administration were oral (61.8%), followed by inhalation (35.5%).

#### CBP source

The source of CBP interventions, whether obtained illicitly, self-prescribed or physician-prescribed, was inadequately documented across the studies, and is therefore not reported.

#### Context of CBP use

Reasons for CBP use also varied considerably, frequently involving multiple conditions within a single study. Nausea and vomiting (51.3%) were identified as the most common reason, followed by cancer pain (24.3%). Only 7.9% of the studies reported participants’ comorbidities.

### Adverse event (AE) characteristics

Table 3 presents the distribution of AEs by CTCAE SOC involving CBP use. Nervous system (77.6%), psychiatric (66.4%) and gastrointestinal AEs (53.3%) were the most common. The majority of studies (80.9%) reported AEs belonging to multiple CTCAE SOCs. Further details are reported in S6.

**Table 3:** Distribution of AEs by CTCAE SOC (*n* = 152)

CTCAE SOC	No. (%)*
Nervous system disorders	118 (77.6%)
Psychiatric disorders	101 (66.4%)
Gastrointestinal disorders	81 (53.3%)
General disorders and administration site conditions	62 (40.8%)
Vascular disorders	43 (28.3%)
Metabolism and nutrition disorders	34 (22.4%)
Eye disorders	32 (21.1%)
Cardiac disorders	31 (20.4%)
Respiratory, thoracic and mediastinal disorders	21 (13.8%)
Death	20 (13.2%)
Skin and subcutaneous tissue disorders	17(11.2%)
Ear and labyrinth disorders	12 (7.9%)
Infections and infestations	12 (7.9%)
Musculoskeletal and connective tissue disorders	12 (7.9%)
Investigations	12 (7.9%)
Blood and lymphatic system disorders	7 (4.6%)
Renal and urinary disorders	7 (4.6%)
Neoplasms benign, malignant and unspecified (incl cysts and polyps)	6 (3.9%)
Drug interactions	4 (2.6%)
Hepatobiliary disorders	3 (2%)
Endocrine disorders	2 (1.3%)
Reproductive system and breast disorders	2 (1.3%)
Congenital, familial and genetic disorders	1 (0.7%)
Immune system disorders	1 (0.7%)
Injury, poisoning and procedural complications	1 (0.7%)

*One hundred twenty-three studies reported AEs in multiple CTCAE SOCs

AEs were inconsistently described across the studies. Sixteen studies applied the CTCAE grading system for reporting severity of the AEs [30, 36, 38, 39, 71, 72, 76, 90, 116, 119, 121, 132, 147, 155, 165, 166]. Other studies employed a combination of serious/non-serious and non-specific grading (e.g. mild/moderate/severe) with many AEs ungraded. Details such as timepoint of AE occurrence, frequency, length of follow-up, clinical outcome, impact on quality of life, AE risk measures and causality were not consistently reported.

AEs were reported by study participants, healthcare practitioners or research staff via questionnaires or surveys conducted through phone calls or in-person visits, health forums and social media platforms.

### Blood and lymphatic system disorders

There were seven studies [72, 73, 87, 92, 134, 155, 166] that reported AEs impacting the blood and lymphatic system. These included anaemia, febrile neutropenia, risk of bleeding and haematological toxicity. Ten studies [29, 40, 72, 86, 90, 92, 103, 134, 151, 166] reported abnormal haematology investigations including decreased neutrophil count, decreased platelet count, low blood count, pancytopenia, prolonged activated partial and thromboplastin time (APTT) associated with oral and inhaled formulations. In general, formulations containing CBD are more commonly implicated in haematological abnormalities [180].

### Cardiac disorders

Thirty-one studies [57–61, 70, 71, 78–80, 89, 90, 95, 99, 100, 115, 120, 126, 132, 150, 159, 163, 168, 174] reported cardiac AEs including secondary myocardial infarction, chest pain, tachycardia, palpitations, bradycardia, cardiovascular event, atrial fibrillation and irregular heartbeat identified with a prolonged electrocardiogram QTc interval. Whilst undefined formulations and those containing various combinations of CBD and THC were associated with cardiac disorders, THC is reported to be the main constituent associated with AEs affecting the cardiovascular system [181].

### Congenital, familial and genetic disorders

Newborn withdrawal syndrome in the first 24 h of life and neonatal peritonitis and intestinal invagination on the 2nd day of life were reported in one neonate whose mother used topical cannabis oil and 1–5 mL pure cannabis oil three times a day at 26 weeks of pregnancy [82].

### Ear and labyrinth disorders

Twelve studies [29, 33, 34, 69, 88, 95, 116, 123–126, 155] reported AEs related to the ear and labyrinth, including vertigo, tinnitus, auditory disorders, ears buzzing, decreased hearing and noise sensitivity.

### Endocrine disorders

Two studies [36, 161] reported AEs affecting the thyroid gland, specifically hypothyroidism and thyroid disorders.

### Eye disorders

Thirty-two studies [29, 33, 34, 43, 46, 47, 52, 53, 56, 61, 67, 78, 79, 88, 89, 92, 95, 98–101, 106, 122, 124–126, 132, 145–147, 150, 166] reported ophthalmic AEs. These included blurred vision, visual distortions, decreased/increased mean intraocular pressure, heavy eyed, ocular swelling and irritation, vision disturbance, amblyopia, visual scotoma, bilateral eye pain, photophobia, eye erythema, swollen eyelids, xerophalmia, dry eyes, vision alterations, itchy eyes, visual floaters and pupil dilation.

### Gastrointestinal disorders

Eighty-one studies [29–31, 33–35, 40, 46–48, 50–53, 56, 58, 59, 61, 65–67, 71, 72, 74, 76, 78–80, 82, 85–90, 95, 97–100, 103–107, 111, 114, 116, 119–125, 127, 130, 132–135, 138, 139, 147, 149–151, 154–156, 159, 161, 163, 166–168, 172–174, 176, 178, 179] reported AEs involving the gastrointestinal system. These included diarrhoea, dry mouth, nausea, vomiting, ascites, cannabinoid hyperemesis syndrome, abdominal pain/cramps/discomfort, sore mouth, GI irritation, constipation, epigastric distress, GERD, oral dysesthesia, persistent CINV, neutropenic colitis with perforation, obstipation, faecal incontinence, aerophagy, gastric ulcer haemorrhage, dysphagia, stomatitis, faecaloma, dyspepsia, mouth ulcers and thirst.

### General disorders and administration site conditions

Sixty-two studies [29–36, 39, 40, 46–48, 52, 57, 61, 66, 72, 75, 76, 78, 81, 83, 85–90, 95, 97–99, 102, 103, 106–108, 114, 122, 127, 132–134, 142, 143, 146–148, 150, 151, 155, 156, 161, 165–169, 171, 172, 178] reported general disorders and administration site AEs including fatigue, distal paresis of arm, pain (general, chest), altered general functioning, general deterioration, deteriorated clinical condition, declining performance status, worsened interference with activities of daily living, inactivity, postural dizziness, physiological side effects, hypothermia, weakness, fever, chills, asthenia, oedema, injection site reaction, local irritation, unsteady feet, unpleasant sensations (related to inhaling cannabis smoke) and gait disturbance.

### Hepatobiliary disorders

Three studies [36, 73, 87] reported hepatobiliary AEs. A case–control study of immunotherapy in cancer [36] documented one case of hepatitis (CTCAE grade ≥ 2) in each group comprising of cannabis users (*n* = 1, 1.5%) and non-users (*n* = 1, 3%). The authors noted that they were classified as immune-related AEs even though relation to immunotherapy was not completely defined. An open-label extension study to investigate the long-term safety and tolerability of CBP in terminal cancer-related pain refractory to strong opioid analgesics [87] reported one case of hepatobiliary disorder in each group using balanced THC/CBD oromucosal spray (*n* = 1, 3%) and THC oromucosal spray (*n* = 1, 25%). There was no information on whether the events were serious, or treatment-related. Increased rates of hepatotoxicity leading to liver injury from CBD-drug interactions were observed in a cross-sectional survey [73] (see “Drug Interactions” section). Studies that reported abnormal liver investigations [86, 90] are also listed in the “Investigations” section.

### Immune system disorders

Immune-related AEs (CTCAE grade ≥ 2) were reported in a case–control study where CBP was used by its participants during immunotherapy [36]. Specific details of these AEs were not disclosed.

### Infections and infestations

Twelve studies [32, 40, 42, 72, 86, 87, 90, 92, 136, 158, 160, 166] reported infections and infestations that included thrush, UTI, pneumonia, invasive pulmonary aspergillosis, oral candidiasis, fungal infection (chest, disseminated), disseminated Fusarium infection, Campylobacter gastroenteritis and lower respiratory tract infection. CBD is the most common constituent observed to be associated with infections [180].

### Injury, poisoning and procedural complications.

An open-label extension study to investigate the long-term safety and tolerability of balanced THC/CBD oromucosal spray and THC oromucosal spray in terminal cancer-related pain refractory to strong opioid analgesics reported AEs in this SOC [87]. Specific details regarding these AEs were not disclosed.

### Investigations

Twelve studies [29, 40, 72, 86, 87, 90, 92, 103, 134, 151, 163, 166] reported AEs identified from investigations, including decreased neutrophil count, decreased platelet count, low blood count, pancytopenia, prolonged activated partial thromboplastin time (APTT), increased gamma-glutamyl transferase (GGT), increased alanine aminotransferase (ALT), increased creatinine, prolonged electrocardiogram QTc interval and weight gain/loss.

### Metabolism and nutrition disorders

Thirty-four studies [31, 34, 35, 40, 43, 46, 56, 63, 76, 78, 80, 86, 87, 90, 93, 103, 106, 114, 125, 126, 130, 132, 134, 146–149, 151, 154, 155, 161, 168, 169, 174] reported AEs related to metabolism and nutritional disorders including appetite increase/loss, anorexia, increased food intake, hunger, dehydration, hyperglycaemia, hypoalbuminemia, hypocalcaemia, hypokalaemia, hypomagnesemia, hyponatremia and hypophosphatemia.

### Musculoskeletal and connective tissue disorders

Twelve studies [33, 34, 43, 52, 87, 95, 125, 132, 147, 150, 161, 166] reported musculoskeletal and connective tissue AEs, including muscle twitching/pain/weakness, leg cramps, limb pain/weakness, arthralgia, back/bone/joint pain, jaw stiffness, decreased motor ability and tremor.

### Neoplasms benign, malignant and unspecified (incl cysts and polyps)

Six studies [86, 87, 103, 134, 155, 166] reported AEs related to neoplasms including tumour progression, tumour-related pain, tumour haemorrhage and metastases to the brain.

### Nervous system disorders

AEs impacting the nervous system were reported in 118 studies [29–35, 38–41, 43, 44, 46–54, 56–63, 65–69, 71–74, 76, 78–80, 83, 85–90, 92–95, 98–100, 102–108, 111, 113, 114, 116, 118–126, 128, 130, 132–134, 137–139, 141–147, 149–157, 159, 162–169, 171, 172, 174–179]. Examples included drowsiness, somnolence, hypersomnia, sedation, lethargy, CNS depression, dizziness, slurred/impaired speech, neuralgia, headache, amnesia, dysgeusia, impaired motor coordination/balance, decreased concentration, paraesthesia, cognitive decline, seizures, syncope, falls, impaired awareness/thinking, cannabinoid-induced alteration of motor-evoked potentials, aphasia and dysarthria. Undefined and defined formulations administered via the oral and inhaled route were associated with AEs involving neurological symptoms. AEs involving the nervous system were mainly attributed to oral and inhaled formulations containing THC, noting some involved combinations of CBD and THC. THC is reported to be the most common constituent associated with AEs affecting the nervous system [181].

### Psychiatric disorders

Psychiatric AEs were reported in 101 studies [28, 29, 31, 33–35, 39, 40, 43–45, 47–53, 56–63, 65–69, 71, 72, 75, 76, 78–80, 83, 85–90, 93–96, 98–100, 103–107, 111, 113, 114, 116, 118–120, 122–128, 131–134, 139, 141, 144–147, 150–152, 154, 156, 157, 159, 163, 166–171, 174–179]. Examples included insomnia, mood disorders (euphoria, feeling “high” or “drunk”, irritability, agitation, dysphoria), hallucination (auditory, visual), panic/fear, anxiety, depression, confusion/disorientation, intoxication, cannabinoid intoxication, psychic disturbance, bulimia, bad/wild/livid dreams, relaxed, depersonalisation/dissociation, distorted perception (time and space), delusions, apathy, paranoia, loss of motivation, psychomimetic effects, hyperventilation, tetanic symptoms, restlessness, personality/behavioural change, psychosis, libido change and hyperactivity. Undefined and defined formulations administered via the oral and inhaled route were associated with psychiatric disorders. However, THC is reported to be the common constituent associated with AEs affecting mental health [182].

### Renal and urinary disorders

Seven studies [47, 86, 87, 90, 147, 166, 167] reported renal and urinary AEs, including urinary retention, cystitis, haematuria and increased nocturia. A phase 1 study of dexanabinol in brain cancer [90] reported treatment-related elevated creatinine (CTCAE grade 1) (*n* = 1, 3.8%) with 36 mg/kg dexanabinol (see the “Investigations” section).

### Reproductive system and breast disorders

Two RCTs involving dronabinol reported AEs involving the reproductive system. One noted a case of vaginal discharge [40] whilst another documented cases of male impotence [85].

### Respiratory, thoracic and mediastinal disorders

Twenty-one studies [33, 35, 40, 48, 61, 73, 76, 87, 92, 98, 99, 116, 117, 127, 147, 154, 155, 163, 166, 171, 178] reported respiratory AEs, most commonly dyspnoea. Others included cough, sore/burning throat, hoarseness, rhinorrhoea, rhinitis, nasal congestion, pneumonitis, respiratory depression and respiratory failure.

### Skin and subcutaneous tissue disorders

Seventeen studies [36, 40, 55, 56, 71, 87, 90, 95, 125, 132, 147, 149, 150, 156, 161, 163, 174] reported AEs involving the skin, such as rash, dry skin, pruritus, urticaria, skin toxicity, hyperhidrosis and alopecia.

### Vascular disorders

Forty-three studies [29, 33, 34, 43, 47, 52, 57–60, 62, 67, 69, 71, 72, 78–80, 83, 86–89, 99, 100, 102–104, 116, 120, 123–126, 132, 133, 138, 139, 150, 152, 154, 156, 167] reported vascular AEs with the most common being hypotension and postural hypotension. Others included hypertension, hot flashes, flushing and a thromboembolic event (pulmonary embolism).

### Drug interactions

Four studies reported CBP-drug interactions. A phase 1 study of dexanabinol in brain cancer [90] reported a case of treatment-related steroid myopathy (CTCAE grade 2) (*n* = 1, 3.8%) with 4 mg/kg dexanabinol. A case report noted the potential inhibition of CYP3A4 and/or CYP2D6 resulting in diminished metabolism of tamoxifen with a low dose CBD of 40 mg/day [129].

A cross-sectional survey by Guedon et al. [73] on CBD use across diverse cancer stages found increased CNS depression was observed in the concomitant use of CBD and morphine, metoclopramide, codeine, fentanyl, tramadol and altered vigilance with cetirizine, levocetirizine, hydroxyzine, alprazolam, quetiapine, zopiclone, clorazepate, sertraline and paroxetine. They also observed that the concomitant use of CBD with oxaliplatin, bortezomib, lenalidomide, dacarbazine, vincristine and methotrexate may lead to an increase in pre-existing hepatotoxicity. Higher rates of hepatotoxicity leading to liver injury were reported with the concomitant use of CBD and sulfamethoxazole-trimethoprim, paracetamol, pravastatin, amiodarone, ramipril or perindopril. Additionally, respiratory depression occurred with morphine, buprenorphine and dexchlorpheniramine whilst increased CBD exposure was noted with aprepitant, ketoconazole and omeprazole. CBD use enhanced exposure to morphine, sirolimus, apixaban and rivaroxaban leading to increased risk of bleeding with rivaroxaban and apixaban. The authors concluded that CBD use posed two primary clinical risks of CNS depression and hepatotoxicity due to drug interactions.

In another cross-sectional survey by Saadeh et al. [143] on medical marijuana use across varying cancer stages, drug interactions were reported with sympathomimetic agents, cytochrome P450 (CYP) isoform 1A2 substrates, CYP2C9 inhibitors and anticholinergic agents, with the highest occurrence noted with CNS depressants.

### Death

Twenty studies [29, 34, 35, 65, 66, 85–87, 92, 103, 110, 127, 134, 142, 148, 155, 158, 165, 166, 173] reported deaths among participants using a CBP. Of these, the authors of ten studies determined that causality was not attributed to CBP use and the remaining did not comment. There were no studies that reported death being directly attributed to CBP use. Further details are provided in S7.

## Discussion

In this review, a wide range of AEs across all CTCAE categories were reported to be associated with a variety of CBP ingredients, forms/types, routes of administration, doses and duration of use, reason for use and patient characteristics. Low-quality reporting and substantial under-reporting of AEs were also identified in many studies. Challenges with synthesising the findings were further compounded by the heterogeneity in the included study designs, as well as the variations in formulations and dosages that often lacked detailed information.

Notwithstanding the limitations that prevented a complete summary of the AEs associated with CBP use, patterns were observed that warrant further research. For instance, many of the nervous system, psychiatric, gastrointestinal and cardiovascular AEs were predictable based on the known pharmacological effects of CBP and its constituents. Some of the AEs involving CNS depression, hepatotoxicity and cardiovascular events could pose serious health risks. These may be reduced with diligent clinical monitoring, careful dosage adjustments and other appropriate precautions [183].

The extensive range of synthetic and natural CBP used, including blends or pure forms of THC and CBD, was noteworthy. The different pharmacological actions of CBP constituents increase the risks of specific AEs. In general, THC is primarily associated with cardiovascular, neurological and psychiatric AEs [181, 182], whilst CBD is implicated in drug interactions, hepatic injury, infections and haematological abnormalities [180]. The diversity in the forms and routes of CBP administration presents a distinct set of potential AEs. Smoking and inhalation can cause respiratory issues including bronchoconstriction and impairment of airway function. Oral ingestion may result in delayed onset of effects and AEs from overuse [184]. The wide variability in doses and duration of CBP use reported may collectively contribute to differences in the types and severity of AEs experienced [185, 186]. Moreover, patients may self-administer CBP at disparate doses and durations based on individual preferences, severity of symptoms and perceived efficacy. Clinicians should consider these variables in the clinical management and monitoring of CBP use to help minimise patient harm. The standardisation of CBP formulations and protocols for use requires urgent attention.

The complex interplay of factors unique to each patient, such as reasons for CBP use, cancer type and stage, genetic variations, comorbidities and concomitant medications, may exert a significant impact on the manifestation of AEs. Patients living with different types of cancer may be susceptible to different AEs related to CBP use due to variations in tumour biology, treatment modalities and systemic effects [186]. The stage of cancer at which CBP are initiated can also influence the development of AEs. Patients with advanced cancer may experience more severe AEs or develop complications that interact with CBP. Genetic polymorphisms can affect the pharmacokinetics and pharmacodynamics of CBP, thereby influencing AE susceptibility and severity [187]. Individual tolerance and response to CBP may also be impacted by other pre-existing medical conditions. This necessitates vigilant monitoring and dose adjustments where required. In addition, the concurrent use of other medications may result in drug interactions with CBP which amplify AEs [188]. Therefore, a personalised approach that accounts for these unique patient characteristics is required.

Challenges with conducting this review included substantial under- or incomplete reporting of AEs. Indeed, 150 studies of CBP use in cancer populations were excluded for this reason. The observed under-reporting of AEs raises important questions about the reliability and completeness of existing data on the safety profile of CBP in cancer care. Improving AE reporting mechanisms is vital for advancing our understanding of CBP safety and promoting evidence-based decision-making in the oncology setting.

This review is not without limitations. Relevant studies may have been missed as grey literature, AE systems and clinical trial databases were omitted. Additionally, we did not contact the authors of potentially relevant excluded studies for further information. A risk of language bias was introduced by limiting to articles in English. An updated search was not conducted, nor did we conduct additional searches for any of the serious AEs reported due to the initial amount of data captured. Notwithstanding, given the large number and breadth of studies included, it is likely that most of the potential AEs associated with CBP use in this population were identified. The exception might be rare AEs that are yet to be reported in a case study or have only been reported in post-marketing surveillance.

Future recommendations include one or more systematic reviews to be undertaken to evaluate the risk of AEs associated with specific CBP and indications. Reviewers should employ critical methodologies to identify, analyse and synthesise existing evidence, providing detailed AE information associated with specific CBP formulations used within an oncology setting. Additionally, researchers should adhere to standardised protocols for reporting clinical studies that include detailed information about the intervention and outcomes.

## Conclusion

The reports included in this review provide preliminary evidence to suggest that AEs are associated with CBP use in the context of cancer care. AEs associated with CBP use were reported across a broad subset of this population and involve a range of defined as well as poorly defined formulations and doses that can impact every body system. The overall inconsistent approaches and standards in the reporting, and under-reporting, of CBP-associated AEs suggest substantial gaps in our knowledge of these AEs that further complicates the assessment of the safety profile of CBP. By addressing these gaps, healthcare providers and policymakers will be better placed to make informed decisions regarding the use of CBP as part of cancer care.

## Supplementary Information

Below is the link to the electronic supplementary material.

Supplementary file1 (PDF 667 KB)

Supplementary file2 (DOCX 21 KB)

Supplementary file3 (DOCX 18 KB)

Supplementary file4 (PDF 443 KB)

Supplementary file5 (PDF 210 KB)

Supplementary file6 (PDF 487 KB)

## Data Availability

No datasets were generated or analysed during the current study.
