# Supplementary material for: Adverse events associated with the use of cannabis-based products in people living with cancer: a systematic scoping review
Source: Support Care Cancer. 2024 Dec 18;33(1):40. doi: 10.1007/s00520-024-09087-w (PMC11655613; doi:10.1007/s00520-024-09087-w)
Supplement: Supplementary file 2 — Supplementary file2 (DOCX 21 KB) [file 520_2024_9087_MOESM2_ESM.docx]

**S2. Search Strategy Example**

Ovid MEDLINE(R) ALL <1946 to May 04, 2023>

1 exp Neoplasms/ 3826287

2 Drug Therapy/ 31162

3 Palliative Care/ or Palliative Medicine/ 62936

4 (neoplas* or cancer* or oncolog* or tumo?r* or malignan* or metasta* or carcinoma* or adenocarcinoma* or choriocarcinoma* or leuk?emia or sarcoma* or teratoma or melanoma or lymphoma or chemo* or palliative* or terminal* or hospice*).mp. 5902737

5 cannabaceae/ or cannabis/ 13467

6 exp Cannabinoids/ 17968

7 Medical Marijuana/ 2192

8 (cannab* or dronabinol* or nabilone* or levonantradol* or tetrahydrocannabinol* or delta-9-THC* or delta-9-tetrahydrocannabinol* or nabiximol* or cesamet* or sativex* or epidiolex* or marijuana* or marihuana* or bhang* or hashish* or ganja* or hemp*).mp. 69059

9 safety/ or patient harm/ or patient safety/ 67323

10 exp Drug Interactions/ 167447

11 Drug-Related Side Effects and Adverse Reactions/ 37562

12 product surveillance, postmarketing/ or adverse drug reaction reporting systems/ or clinical trials, phase iv as topic/ or pharmacovigilance/ or prescription drug monitoring programs/ 18114

13 (safety* or harm* or interaction* or pharmacovigilance*).mp. 2673851

14 ((adverse* or side*) adj3 (reaction* or effect* or outcome* or event*)).mp. 2580589

15 1 or 2 or 3 or 4 6125687

16 5 or 6 or 7 or 8 69059

17 9 or 10 or 11 or 12 or 13 or 14 4921249

18 15 and 16 and 17 2157
