# Supplementary material for: Adverse events associated with the use of cannabis-based products in people living with cancer: a systematic scoping review
Source: Support Care Cancer. 2024 Dec 18;33(1):40. doi: 10.1007/s00520-024-09087-w (PMC11655613; doi:10.1007/s00520-024-09087-w)
Supplement: Supplementary file 3 — Supplementary file3 (DOCX 18 KB) [file 520_2024_9087_MOESM3_ESM.docx]

**S3. Search terms used in search strategy**

| **Population:**  **Cancer Diagnosis** | **Concept 1:**  **Adverse Effects** | **Concept 2:**  **Cannabis-based Products** |
| --- | --- | --- |
| neoplasm | adverse effect | cannabis |
| cancer | adverse reaction | cannabaceae |
| oncolog* | adverse event | cannabinoid |
| tumo?r* | adverse outcome | dronabinol |
| malignan* | side effect | nabilone |
| metasta* | safety | levonantradol |
| carcinoma | harm | tetrahydrocannabinol |
| adenocarcinoma | interaction | delta-9-THC |
| choriocarcinoma | pharmacovigilance | delta-9-tetrahydrocannabinol |
| leuk?emia |  | nabiximol |
| sarcoma |  | cesamet |
| teratoma |  | sativex |
| melanoma |  | epidiolex |
| lymphoma |  | marijuana |
| chemo* |  | marihuana |
| palliative* |  | bhang |
| terminal* |  | hashish |
| hospice* |  | ganja |
|  |  | hemp |
