# Supplementary material for: Adverse events associated with the use of cannabis-based products in people living with cancer: a systematic scoping review
Source: Support Care Cancer. 2024 Dec 18;33(1):40. doi: 10.1007/s00520-024-09087-w (PMC11655613; doi:10.1007/s00520-024-09087-w)
Supplement: Supplementary file 4 — Supplementary file4 (PDF 443 KB) [file 520_2024_9087_MOESM4_ESM.pdf]

S4. Data Extraction Template

ARTICLE INFORMATION

Study ID (e.g. Last Name, Year)

Citation details

Country of study

(select all that apply)

1. Australia
2. Canada
3. Israel
4. New Zealand
5. UK
6. US
7. Other

DECLARATIONS

Was funding and interests declared?

1. Funding & interests declared
2. Funding declared; interests not declared
3. Interests declared; funding not declared
4. Not declared

Details if funding declared

(write: declared no funding or provide details)

Details if interests declared

(write: declared no interest or provide details)

STUDY CHARACTERISTICS

Study design

1. Randomised controlled trial
2. Non-randomised controlled trial
3. Case-control study
4. Longitudinal cohort study
5. Cross-sectional survey
6. Retrospective chart review
7. Single-arm observational study (prospective)
8. Case series
9. Case report
10. Other

Indication of CBP or condition studied for cancer population

(select all that apply)

1. General/cancer care (non-specific symptoms)
2. Anorexia
3. Cachexia
4. Cancer pain
5. Fatigue
6. Insomnia
7. Mental health (anxiety, depression etc)
8. Nausea and vomiting
9. Neuropathy
10. Seizures
11. Other

PATIENT CHARACTERISTICS

Number of participants on CBP

(number who received at least 1 dose of CBP)

Number of participants in comparison group

Age group

(select all that apply)

1. Infant (below 1 year)

2. Child (1-17 years)
3. Adult (18-64 years)
4. Elderly (65 years and above)
5. Unknown

Gender

(select all that apply)

1. Not reported
2. Men/boys
3. Women/girls
4. Other

Ethnicity

1. Not reported
2. Other

Cancer diagnosis

(select all that apply)

1. Not reported
2. General
3. Breast
4. Endocrine
5. Gastrointestinal, liver
6. Gynaecological
7. Haematological
8. Head/neck
9. Lung
10. Neurological
11. Skin
12. Urogenital
13. Other

Cancer stage

1. Not reported
2. Mixed
3. Early
4. Advanced

Comorbidities

1. Not reported
2. No
3. Yes (details not provided)
4. Yes

Details about comorbidities

Prior experience with CBP

1. Not reported
2. Yes
3. No
4. Mixed
5. Other

Exclusion criteria of populations with an increased risk of AEs:

(select all that apply)

1. N/A
2. Not reported
3. Neurological
4. Psychiatric
5. Cardiovascular
6. History or current drug/alcohol/tobacco use
7. Renal
8. Hepatic
9. Other

INTERVENTIONS

CBP Intervention

CBP ingredient

(select all that apply)

1. Not reported

2. THC
3. Delta-8-THC
4. CBD
5. High THC:CBD
6. High CBD:THC
7. Balanced THC: CBD
8. Dronabinol
9. Levonantradol
10. Nabilone
11. Sativex
12. Other

CBP form/type

(select all that apply)

1. Not reported
2. Bud
3. Inflorescence/flower
4. Plant
5. Capsule
6. Cigarette
7. Oil
8. Oral solution
9. Spray
10. Syrup
11. Tablet
12. Tincture
13. Topical agent
14. Injection
15. Infusion
16. Other

CBP route of administration

(select all that apply)

1. Oral
2. Inhaled/vaporised

3. Topical
4. Enteral
5. Oromucosal
6. Smoking
7. Sublingual
8. IM
9. IV
10. Other

CBP dose

CBP duration of use

Details about CBP intervention

Comparator Intervention

Comparator ONE ingredient

(select all that apply)

1. Not reported
2. None
3. Placebo
4. Chlorpromazine
5. Dexamethasone
6. Dimenhydrinate
7. Diphenhydramine
8. Domperidone
9. Levomepromazine
10. Levonantradol
11. Metoclopramide
12. Prochlorperazine
13. Thiethylperazine
14. Triflupromazine
15. Other

Comparator ONE form/type

(select all that apply)

1. Capsule
2. Infusion
3. Injection
4. Patch
5. Solution
6. Tablet
7. Other

Comparator ONE route of administration

(select all that apply)

1. Oral
2. IM
3. IV
4. Topical
5. Other

Comparator ONE dose

Comparator ONE duration of use

Comparator TWO ingredient

1. Placebo
2. Chlorpromazine
3. Dexamethasone
4. Diphenhydramine
5. Domperidone
6. Levonantradol
7. Metoclopramide
8. Prochlorperazine
9. Thiethylperazine
10. Other

Comparator TWO form/type

1. Capsule
2. Infusion
3. Injection
4. Patch
5. Solution

6. Tablet
7. Other

Comparator TWO route of administration

1. Oral
2. IM
3. IV
4. Topical
5. Other

Comparator TWO dose

Comparator TWO duration of use

Details about comparator intervention

Concomitant Intervention

Concomitant Intervention

(select all that apply)

1. Not reported
2. None
3. Biological
4. Chemotherapy
5. Radiotherapy
6. Immunotherapy
7. Hormone therapy
8. Opioids
9. Other analgesics
10. Corticosteroids
11. Antiemetics
12. Antiepileptics
13. Pyschotropics
14. Cardiovascular
15. Other

Details about concomitant intervention

	Ingredients	Dosage Regimen
1		
2		
3		
4		
5		
6		
7		
8		

Details about recreational drug/tobacco/alcohol use

1. Not reported
2. None
3. Other

ADVERSE EVENTS

How AEs were reported

(select all that apply)

1. HCP (medical practitioner/physician/nurse)
2. Participant
3. Research staff (investigator/trial nurse)
4. Questionnaire
5. Phone call
6. Visit
7. Other

How AEs were graded

(select all that apply)

1. Not graded
2. CTCAE grading
3. Non-serious/serious
4. Non-specific (e.g. mild/moderate/severe)
5. Other

Details about AEs

	AE	Number of AEs	Number of patients with AE (% patients)	Total number of patients	Category and severity (CTCAE grading)	Timepoint of occurrence after commencement of CBP	Outcome (including withdrawal/discontinuation, impact on QoL due to AEs)	Other details (e.g. patient details, causality, risk of AE e.g. RR, AR, OR)
1								
2								
3								
4								
5								
6								
7								
8								
9								

	AE	Number of AEs	Number of patients with AE (% patients)	Total number of patients	Category and severity (CTCAE grading)	Timepoint of occurrence after commencement of CBP	Outcome (including withdrawal/discontinuation, impact on QoL due to AEs)	Other details (e.g. patient details, causality, risk of AE e.g. RR, AR, OR)
10								
11								
12								
13								
14								
15								
16								
17								
18								
19								
20								

If more than 20 AEs – write details here

Further details about AEs

AUTHOR'S CONCLUSION
