# Supplementary material for: Adverse events associated with the use of cannabis-based products in people living with cancer: a systematic scoping review
Source: Support Care Cancer. 2024 Dec 18;33(1):40. doi: 10.1007/s00520-024-09087-w (PMC11655613; doi:10.1007/s00520-024-09087-w)
Supplement: Supplementary file 5 — Supplementary file5 (PDF 210 KB) [file 520_2024_9087_MOESM5_ESM.pdf]

S5. Excluded Articles

Authors	Title	Journal	Published Year	Volume	Issue	Pages	DOI	Exclusion Reason
Nathan, Rachel; Mupamombe, Charles T; Elibol, John; Case, Amy A; Smith, Danielle; Hyland, Andrew; Attwood, Kristopher; Hansen, Eric D	Assessing Efficacy and Use Patterns of Medical Cannabis for Symptom Management in Elderly Cancer Patients.	The American journal of hospice & palliative care	2023	40	4	368-373	https://dx.doi.org/10.1177/10499091221110217	No information about AEs for cancer population.
Hallinan, Christine M; Gunn, Jane M; Bonomo, Yvonne A	Use of electronic medical records to monitor the safe and effective prescribing of medicinal cannabis: is it feasible?.	Australian journal of primary health	2022	28	6	564-572	https://dx.doi.org/10.1071/PY22054	No information about AEs for cancer population.
Assanangkornchai, Sawitri; Thaikla, Kanittha; Talek, Muhammadfahmee; Saingam, Darika	Medical cannabis use in Thailand after its legalization: a respondent-driven sample survey.	PeerJ	2022	10	101603425	e12809	https://dx.doi.org/10.7717/peerj.12809	No information about AEs for cancer population.
HaGani, Neta; Sznitman, Sharon; Dor, Michael; Bar-Sela, Gil; Oren, Dana; Margolis-Dorfman, Lilia; Goor-Aryeh, Itay; Green, Manfred S	Attitudes Toward the Use of Medical Cannabis and the Perceived Efficacy, Side-effects and Risks: A Survey of Patients, Nurses and Physicians.	Journal of psychoactive drugs	2022	54	5	393-402	https://dx.doi.org/10.1080/02791072.2021.2009598	No information about AEs for cancer population.
Kalonji, Pauline; Revol, Aurelie; Broers, Barbara; Ljuslin, Michael; Pautex, Sophie	Patient-Related Barriers to the Prescription of Cannabinoid-Based Medicines in Palliative Care: A Qualitative Approach.	Palliative medicine reports	2022	3	1	200-205	https://dx.doi.org/10.1089/pmr.2022.0021	No information about AEs for cancer population.
Laborada, Jennifer; Cohen, Philip R	Cutaneous Squamous Cell Carcinoma and Lichen Simplex Chronicus Successfully Treated with Topical Cannabinoid Oil: A Case Report and Summary of Cannabinoids in Dermatology.	Cureus	2022	14	4	e23850	https://dx.doi.org/10.7759/cureus.23850	Wrong study design (not primary data).
Bar-Lev Schleider, Lihi; Mechoulam, Raphael; Sikorin, Inbal; Naftali, Timna; Novack, Victor	Adherence, Safety, and Effectiveness of Medical Cannabis and Epidemiological Characteristics of the Patient Population: A Prospective Study.	Frontiers in medicine	2022	9	101648047	827849	https://dx.doi.org/10.3389/fmed.2022.827849	No information about AEs for cancer population.
Nimalan, Devaki; Kawka, Michal; Erridge, Simon; Ergisi, Mehmet; Harris, Michael; Salazar, Oliver; Ali, Rayyan; Loupasaki, Katerina; Holvey, Carl; Coomber, Ross; Platt, Michael; Rucker, James J; Khan, Shaheen; Sodergren, Mikael H	UK Medical Cannabis Registry palliative care patients cohort: initial experience and outcomes.	Journal of cannabis research	2022	4	1	3	https://dx.doi.org/10.1186/s42238-021-00114-9	No information about AEs for cancer population.
Meghani, Salimah H; Quinn, Ryan; Ashare, Rebecca; Levoy, Kristin; Worster, Brooke; Naylor, Mary; Chittams, Jesse; Cheatle, Martin	Impact of Cannabis Use on Least Pain Scores Among African American and White Patients with Cancer Pain: A Moderation Analysis.	Journal of pain research	2021	14	101540514	3493-3502	https://dx.doi.org/10.2147/JPR.S332447	No information about AEs for cancer population.
Waissengrin, Barliz; Mirelman, Dan; Pelles, Sharon; Bukstein, Felix; Blumenthal, Deborah T; Wolf, Ido; Geva, Ravit	Effect of cannabis on oxaliplatin-induced peripheral neuropathy among oncology patients: a retrospective analysis.	Therapeutic advances in medical oncology	2021	13	101510808	1758835921990203	https://dx.doi.org/10.1177/1758835921990203	No information about AEs for cancer population.
Gulbransen, Graham; Xu, William; Arroll, Bruce	Cannabidiol prescription in clinical practice: an audit on the first 400 patients in New Zealand.	BJGP open	2020	4	1		https://dx.doi.org/10.3399/bjgpopen20X101010	No information about AEs for cancer population.
Wongkongdech, Ranee; Pansila, Narisara; Nonetoom, Pichit; Turnbull, Niruwan; Wongkongdech, Adisorn	Symptom Management and Quality of Life of Palliative Cancer Patients After Being Administered with Thai Medicinal Cannabis.	Studies in health technology and informatics	2022	295	ck1, 9214582	450-453	https://dx.doi.org/10.3233/SHTI220762	No information about AEs for cancer population.
Weiss, Marisa C; Hibbs, Julianne E; Buckley, Meghan E; Danese, Sherry R; Leitenberger, Adam; Bollmann-Jenkins, Melissa; Meske, Sam W; Aliano-Ruiz, Katherine E; McHugh, Theresa W; Larson, Sharon L; Le, Elaine H; Green, Nancye L; Gilman, Paul B; Kaklamani, Virginia G; Chlebowski, Rowan T; Martinez, Diana M	A Coala-T-Cannabis Survey Study of breast cancer patients' use of cannabis before, during, and after treatment.	Cancer	2022	128	1	160-168	https://dx.doi.org/10.1002/cncr.33906	No information about AEs for cancer population.
Russo, Ethan B; Cuttler, Carrie; Cooper, Ziva D; Stueber, Amanda; Whiteley, Venetia L; Sexton, Michelle	Survey of Patients Employing Cannabigerol-Predominant Cannabis Preparations: Perceived Medical Effects, Adverse Events, and Withdrawal Symptoms.	Cannabis and cannabinoid research	2022	7	5	706-716	https://dx.doi.org/10.1089/can.2021.0058	No information about AEs for cancer population.

Authors	Title	Journal	Published Year	Volume	Issue	Pages	DOI	Exclusion Reason
Wiseman, Luke K; Mahu, Ioan T; Mukhida, Karim	The Effect of Preoperative Cannabis Use on Postoperative Pain Following Gynaecologic Oncology Surgery.	Journal of obstetrics and gynaecology Canada : JOGC = Journal d'obstetrique et gynecologie du Canada : JOGC	2022	44	7	750-756	https://dx.doi.org/10.1016/j.jogc.2022.01.018	Wrong concept (study reporting/evaluating the risk of developing cancer associated with CBP use in other populations).
Tumati, Shankar; Lanctot, Krista L; Wang, RuoDing; Li, Abby; Davis, Andrew; Herrmann, Nathan	Medical Cannabis Use Among Older Adults in Canada: Self-Reported Data on Types and Amount Used, and Perceived Effects.	Drugs & aging	2022	39	2	153-163	https://dx.doi.org/10.1007/s40266-021-00913-y	No information about AEs for cancer population.
Divisic, Antuan; Avagnina, Irene; De Tommasi, Valentina; Santini, Anna; Brogelli, Laura; Giacomelli, Luca; Benini, Franca	The use of medical cannabis in pediatric palliative care: a case series.	Italian journal of pediatrics	2021	47	1	229	https://dx.doi.org/10.1186/s13052-021-01179-1	No information about AEs for cancer population.
McTaggart-Cowan, Helen; Bentley, Colene; Raymakers, Adam; Metcalfe, Rebecca; Hawley, Philippa; Peacock, Stuart	Understanding cancer survivors' reasons to medicate with cannabis: A qualitative study based on the theory of planned behavior.	Cancer medicine	2021	10	1	396-404	https://dx.doi.org/10.1002/cam4.3536	No information about AEs for cancer population.
Hawley, Philippa; Gobbo, Monica; Afghari, Narsis	The impact of legalization of access to recreational Cannabis on Canadian medical users with Cancer.	BMC health services research	2020	20	1	977	https://dx.doi.org/10.1186/s12913-020-05756-8	No information about AEs for cancer population.
Hardy, Janet; Haywood, Alison; Gogna, Gauri; Martin, Jennifer; Yates, Patsy; Greer, Ristan; Good, Phillip	Oral medicinal cannabinoids to relieve symptom burden in the palliative care of patients with advanced cancer: a double-blind, placebo-controlled, randomised clinical trial of efficacy and safety of 1:1 delta-9-tetrahydrocannabinol (THC) and cannabidiol (	Trials	2020	21	1	611	https://dx.doi.org/10.1186/s13063-020-04541-6	Wrong study design (not primary data).
Anamnart, Chumpol; Jitprapaikulsan, Jiraporn	Anti-LGI1 encephalitis in a middle-aged woman who consumes cannabis oil.	Neurological sciences : official journal of the Italian Neurological Society and of the Italian Society of Clinical Neurophysiology	2020	41	12	3755-3757	https://dx.doi.org/10.1007/s10072-020-04497-0	No information about AEs for cancer population.
Lintzeris, Nicholas; Mills, Llewellyn; Suraev, Anastasia; Bravo, Maria; Arkell, Thomas; Arnold, Jonathon C; Benson, Melissa J; McGregor, Iain S	Medical cannabis use in the Australian community following introduction of legal access: the 2018-2019 Online Cross-Sectional Cannabis as Medicine Survey (CAMS-18).	Harm reduction journal	2020	17	1	37	https://dx.doi.org/10.1186/s12954-020-00377-0	No information about AEs for cancer population.
Podda, Marta; Pagani Bagliacca, Elena; Sironi, Giovanna; Veneroni, Laura; Silva, Matteo; Angi, Martina; Massimino, Maura; Ferrari, Andrea; Clerici, Carlo Alfredo	Cannabinoids use in adolescents and young adults with cancer: a single-center survey.	Tumori	2020	106	4	281-285	https://dx.doi.org/10.1177/0300891620912022	No information about AEs for cancer population.
Highet, Bridget H; Lesser, Elizabeth R; Johnson, Patrick W; Kaur, Judith S	Tetrahydrocannabinol and Cannabidiol Use in an Outpatient Palliative Medicine Population.	The American journal of hospice & palliative care	2020	37	8	589-593	https://dx.doi.org/10.1177/1049909119900378	No information about AEs for cancer population.
Zarrabi, Ali John; Welsh, Justine W; Sniecinski, Roman; Curseen, Kimberly; Gillespie, Theresa; Baer, Wendy; McKenzie-Brown, Anne Marie; Singh, Vinita	Perception of Benefits and Harms of Medical Cannabis among Seriously Ill Patients in an Outpatient Palliative Care Practice.	Journal of palliative medicine	2020	23	4	558-562	https://dx.doi.org/10.1089/jpm.2019.0211	No information about AEs for cancer population.
Donovan, Kristine A; Oberoi-Jassal, Ritika; Chang, Young D; Rajasekhara, Sahana; Haas, Meghan F; Randich, Anthony L; Portman, Diane G	Cannabis Use in Young Adult Cancer Patients.	Journal of adolescent and young adult oncology	2020	9	1	30-35	https://dx.doi.org/10.1089/jayao.2019.0039	No information about AEs for cancer population.
Boland, Diane M; Reidy, Lisa J; Seither, Joshua M; Radtke, Jay M; Lew, Emma O	Forty-Three Fatalities Involving the Synthetic Cannabinoid, 5-Fluoro-ADB: Forensic Pathology and Toxicology Implications.	Journal of forensic sciences	2020	65	1	170-182	https://dx.doi.org/10.1111/1556-4029.14098	No information about AEs for cancer population.
Vulfsons, Simon; Ognitz, Miriam; Bar-Sela, Gil; Raz-Pasteur, Ayelet; Eisenberg, Elon	Cannabis treatment in hospitalized patients using the SYQE inhaler: Results of a pilot open-label study.	Palliative & supportive care	2020	18	1	Dec-17	https://dx.doi.org/10.1017/S147895151900021X	No information about AEs for cancer population.
Lamba, Nayan; Mahal, Brandon A; Martinez, Ruben; Leland, Peggy; Shih, Helen A	Radiation Therapy Pain Management: Prevalence of Symptoms and Effectiveness of Treatment Options.	Clinical journal of oncology nursing	2019	23	5	514-521	https://dx.doi.org/10.1188/19.CJON.514-521	No information about AEs for cancer population.

Authors	Title	Journal	Published Year	Volume	Issue	Pages	DOI	Exclusion Reason
Bialas, Patric; Drescher, Beate; Gottschling, Sven; Juckenhofel, Stephanie; Konietzke, Dieter; Kuntz, Wolfgang; Kuhne-Adler, Isabell; Merl-Ripplinger, Heidi; Preisegger, Diether; Schneider, Kathrein; Straus, Manfred; Welsch, Patrick; Hauser, Winfried	[Cannabis-based medicines for chronic pain: indications, selection of drugs, effectiveness and safety : Experiences of pain physicians in Saarland].	Schmerz (Berlin, Germany)	2019	33	5	399-406	https://dx.doi.org/10.1007/s00482-019-0383-1	Non-English language article.
Wilson, Matthew McEwen; Masterson, Emily; Broglio, Kathleen	Cannabis Use among Patients in a Rural Academic Palliative Care Clinic.	Journal of palliative medicine	2019	22	10	1224-1226	https://dx.doi.org/10.1089/jpm.2018.0534	No information about AEs for cancer population.
Steele, Grant; Arneson, Tom; Zylla, Dylan	A Comprehensive Review of Cannabis in Patients with Cancer: Availability in the USA, General Efficacy, and Safety.	Current oncology reports	2019	21	1	10	https://dx.doi.org/10.1007/s11912-019-0757-7	Wrong study design (not primary data).
Martell, K; Fairchild, A; LeGerrier, B; Sinha, R; Baker, S; Liu, H; Ghose, A; Olivotto, I A; Kerba, M	Rates of cannabis use in patients with cancer.	Current oncology (Toronto, Ont.)	2018	25	3	219-225	https://dx.doi.org/10.3747/co.25.3983	No information about AEs for cancer population.
Abuhasira, Ran; Schleider, Lihi Bar-Lev; Mechoulam, Raphael; Novack, Victor	Epidemiological characteristics, safety and efficacy of medical cannabis in the elderly.	European journal of internal medicine	2018	49	9003220	44-50	https://dx.doi.org/10.1016/j.ejim.2018.01.019	No information about AEs for cancer population.
Ozturk, Hayriye M; Erdogan, Mehmet; Alsancak, Yakup; Yarlioglues, Mikail; Duran, Mustafa; Boztas, M Hamid; Murat, Sani N; Ozturk, Selcuk	Electrocardiographic alterations in patients consuming synthetic cannabinoids.	Journal of psychopharmacology (Oxford, England)	2018	32	3	296-301	https://dx.doi.org/10.1177/0269881117736918	No information about AEs for cancer population.
Vin-Raviv, Neomi; Akinyemiju, Tomi; Meng, Qingrui; Sakhuja, Swati; Hayward, Reid	Marijuana use and inpatient outcomes among hospitalized patients: analysis of the nationwide inpatient sample database.	Cancer medicine	2017	6	1	320-329	https://dx.doi.org/10.1002/cam4.968	Wrong concept (study reporting/evaluating the risk of developing cancer associated with CBP use in other populations).
Fanelli, Guido; De Carolis, Giuliano; Leonardi, Claudio; Longobardi, Adele; Sarli, Ennio; Allegri, Massimo; Schatman, Michael E	Cannabis and intractable chronic pain: an explorative retrospective analysis of Italian cohort of 614 patients.	Journal of pain research	2017	10	101540514	1217-1224	https://dx.doi.org/10.2147/JPR.S132814	No information about AEs for cancer population.
Zolotov, Yuval; Baruch, Yehuda; Reuveni, Haim; Magnezi, Racheli	Adherence to Medical Cannabis Among Licensed Patients in Israel.	Cannabis and cannabinoid research	2016	1	1	16-21	https://dx.doi.org/10.1089/can.2015.0003	No information about AEs for cancer population.
Elder, Joshua J; Knoderer, Holly M	Characterization of Dronabinol Usage in a Pediatric Oncology Population.	The journal of pediatric pharmacology and therapeutics : JPPT : the official journal of PPAG	2015	20	6	462-7	https://dx.doi.org/10.5863/1551-6776-20.6.462	No information about AEs for cancer population.
Aldington, S; Harwood, M; Cox, B; Weatherall, M; Beckert, L; Hansell, A; Pritchard, A; Robinson, G; Beasley, R	Cannabis use and risk of lung cancer: a case-control study.	The European respiratory journal	2008	31	2	280-6	https://dx.doi.org/10.1183/09031936.00065707	Wrong concept (study reporting/evaluating the risk of developing cancer associated with CBP use in other populations).
Gorter, Robert W; Butorac, Mario; Cobian, Eloy Pulido; van der Sluis, Willem	Medical use of cannabis in the Netherlands.	Neurology	2005	64	5	917-9		No information about AEs for cancer population.
Schwartz, R H; Beveridge, R A	Marijuana as an antiemetic drug: how useful is it today? Opinions from clinical oncologists.	Journal of addictive diseases	1994	13	1	53-65		Hearsay/opinion on AEs.
Sacks, N; Hutcheson, J R Jr; Watts, J M; Webb, R E	Case report: the effect of tetrahydrocannabinol on food intake during chemotherapy.	Journal of the American College of Nutrition	1990	9	6	630-2		No information about AEs for cancer population.
Few, B J	Nabilone as an antiemetic for children undergoing chemotherapy.	MCN. The American journal of maternal child nursing	1988	13	3	209		Wrong study design (not primary data).
Ward, A; Holmes, B	Nabilone. A preliminary review of its pharmacological properties and therapeutic use.	Drugs	1985	30	2	127-44		Wrong study design (not primary data).
George, M; Pejovic, M H; Thuaire, M; Kramar, A; Wolff, J P	[Randomized comparative trial of a new anti-emetic: nabilone, in cancer patients treated with cisplatin].	Biomedicine & pharmacotherapy = Biomedecine & pharmacotherapie	1983	37	1	24-Jul		Non-English language article.
Heim, M E; Queisser, W	[Treatment of refractory cytostatic agent-induced vomiting with the synthetic cannabinoid levonantradol].	Onkologie	1982	5	2	94-6		Non-English language article.
Cocchetto, D M; Cook, L F; Cato, A E	A critical review of the safety and antiemetic efficacy of delta-9-tetrahydrocannabinol.	Drug intelligence & clinical pharmacy	1981	15	11	867-75		Unable to obtain full text.
Williams, C J; Bolton, A; de Pemberton, R; Whitehouse, J M	Antiemetics for patients treated with antitumor chemotherapy.	Cancer clinical trials	1980	3	4	363-7		No information about AEs for cancer population.

Authors	Title	Journal	Published Year	Volume	Issue	Pages	DOI	Exclusion Reason
Kawka M.; Erridge S.; Holvey C.; Coomber R.; Usmani A.; Sajad M.; Platt M.W.; Rucker J.J.; Sodergren M.H.	Clinical outcome data of first cohort of chronic pain patients treated with cannabis-based sublingual oils in the United Kingdom - Analysis from the UK Medical Cannabis Registry	European Journal of General Practice	2023	29	1	9	https://dx.doi.org/10.1080/13814788.2023.2171393	Meeting abstracts on RCTs/non-RCTs/surveys/case series or case studies with very low quality non-specific AE reporting.
Segar J.; Farr K.; Junak M.; Roe D.; Ehsani S.; Jiralerspong S.; Mohab I.; Vanderah T.; Chalasani P.	Evaluation of Dronabinol to Decrease Opioid Use for Cancer- Induced Bone Pain	Cancer Research	2023	83	5 Supplement		https://dx.doi.org/10.1158/1538-7445.SABCS22-P4-04-02	Meeting abstracts on RCTs/non-RCTs/surveys/case series or case studies with very low quality non-specific AE reporting.
Horsted T.; Hesthaven K.L.; Leutscher P.D.C.	Safety and effectiveness of cannabinoids to Danish patients with treatment refractory chronic pain-A retrospective observational real-world study	European Journal of Pain (United Kingdom)	2023	27	2	234-247	https://dx.doi.org/10.1002/ejp.2054	No information about AEs for cancer population.
Keyhani S.; Leonard S.; Byers A.L.; Zaman T.; Krebs E.; Austin P.C.; Moss-Vazquez T.; Austin C.; Sandbrink F.; Bravata D.M.	Association of a Positive Drug Screening for Cannabis with Mortality and Hospital Visits among Veterans Affairs Enrollees Prescribed Opioids	JAMA Network Open	2022	5	12	E2247201	https://dx.doi.org/10.1001/jamanetworkopen.2022.47201	No information about AEs for cancer population.
Rombouts M.; Karg R.; Crul M.	A DESCRIPTIVE, OBSERVATIONAL AND CROSSSECTIONAL STUDY TO DETERMINE THE PREVALENCE AND RISKS OF COMPLEMENTARY AND ALTERNATIVE MEDICINE USE IN AN ADULT DUTCH (HEMATO)-ONCOLOGY POPULATION RECEIVING CONCURRENT ANTI-CANCER TREATMENT	European Journal of Oncology Pharmacy	2023	6	1 Supplement	16	https://dx.doi.org/10.1097/OP9.0000000000000042	Meeting abstracts on RCTs/non-RCTs/surveys/case series or case studies with very low quality non-specific AE reporting.
Ueberall M.A.; Horlemann J.; Schuermann N.; Kalaba M.; Ware M.A.	Effectiveness and Tolerability of Dronabinol Use in Patients with Chronic Pain: A Retrospective Analysis of 12-Week Open-Label Real-World Data Provided by the German Pain e-Registry	Pain Medicine (United States)	2022	23	8	1409-1422	https://dx.doi.org/10.1093/pm/pnac010	No information about AEs for cancer population.
Massalha H.; Tocut M.; Stein M.; Zandman-Goddard G.	Hypereosinophilia and Paraneoplastic Syndrome: An Unusual Presentation with an Unexpected Diagnosis	Israel Medical Association Journal	2022	24	11	779-780		Wrong concept (study reporting/evaluating the risk of developing cancer associated with CBP use in other populations).
Wanishpongpan S.; Oranratanaphan S.	A RANDOMIZED CONTROLLED STUDY BETWEEN THC CANNABIS OIL AND PLACEBO ADDED ON STANDARD PROPHYLAXIS FOR REDUCING CHEMOTHERAPY-INDUCED NAUSEA VOMITING (CINV) FOLLOWING CARBOPLATIN AND PACLITAXEL REGIMEN	International Journal of Gynecological Cancer	2022	32	Supplement 3	A216	https://dx.doi.org/10.1136/ijgc-2022-igcs.490	Meeting abstracts on RCTs/non-RCTs/surveys/case series or case studies with very low quality non-specific AE reporting.
Zarrabi A.J.; Welsh J.W.; Sniecinski R.; Curseen K.; Gillespie T.; Baer W.; McKenzie-Brown A.M.; Singh V.	Perception of Benefits and Harms of Medical Cannabis among Seriously Ill Patients in an Outpatient Palliative Care Practice	Journal of palliative medicine	2019		(Zarrabi, Curseen) Department of Family and Preventive Medicine, Emory University, Atlanta, Georgia(Welsh, Baer) Department of Psychiatry and Behaviorial Sciences, Emory University, Atlanta, Georgia(Sniecinski, McKenzie-Brown, Singh) Department of Anesthe		https://dx.doi.org/10.1089/jpm.2019.0211	No information about AEs for cancer population.

Authors	Title	Journal	Published Year	Volume	Issue	Pages	DOI	Exclusion Reason
Donovan K.A.; Oberoi-Jassal R.; Chang Y.D.; Rajasekhara S.; Haas M.F.; Randich A.L.; Portman D.G.	Cannabis Use in Young Adult Cancer Patients	Journal of adolescent and young adult oncology	2019		(Donovan, Oberoi-Jassal, Chang, Rajasekhara, Haas, Randich, Portman) Department of Supportive Care Medicine, Moffitt Cancer Center, Tampa, FL, United States		https://dx.doi.org/10.1089/jayao.2019.0039	No information about AEs for cancer population.
Ashare R.; Turay E.; Worster B.; Meghani S.	Social Determinants of Health Associated With How Cannabis is Obtained and Used in Patients With Cancer	Neuropsychopharmacology	2022	47	Supplement 1	462	https://dx.doi.org/10.1038/s41386-022-01486-z	No information about AEs for cancer population.
Trojan A.; Breitkopf S.; Pittl S.; Heeren M.	SwissCanOn - scientific patient registry for medicinal cannabis in oncology including ePROs - Trial in Progress	Swiss Medical Weekly	2022	152	265	50S		Meeting abstracts on RCTs/non-RCTs/surveys/case series or case studies with very low quality non-specific AE reporting.
Lakhani S.; Scalzitti D.A.; Padrone L.; Martins-Welch D.	From evidence to practice: early integration of palliative care in a comprehensive cancer center	Supportive Care in Cancer	2023	31	1	17	https://dx.doi.org/10.1007/s00520-022-07510-8	No information about AEs for cancer population.
Nathan R.A.; Tonderai C.; Mupamombe; Walter M.; Case A.A.; Hansen E.	Use of medical cannabis in treating anorexia and nausea in elderly cancer patients	Journal of Clinical Oncology	2019	37	31 Supplement 1		https://dx.doi.org/10.1200/JCO.2019.37.31_suppl.124	Meeting abstracts on RCTs/non-RCTs/surveys/case series or case studies with very low quality non-specific AE reporting.
Donovan K.A.	Age-related differences in cannabis use by cancer patients referred for supportive care. Diane Portman	Journal of Clinical Oncology	2019	37	31 Supplement 1		https://dx.doi.org/10.1200/JCO.2019.37.31_suppl.104	Meeting abstracts on RCTs/non-RCTs/surveys/case series or case studies with very low quality non-specific AE reporting.
Cantor E.; Meyer A.; Morris S.M.; Weisenberg J.L.Z.; Brossier N.M.	Dose-dependent seizure control with MEK inhibitor therapy for progressive glioma in a child with neurofibromatosis type 1	Child's Nervous System	2022	38	11	2245-2249	https://dx.doi.org/10.1007/s00381-022-05571-y	No information about AEs for cancer population.
Zurcher K.; Dupont C.; Weber P.; Grunt S.; Wilhelm I.; Eigenmann D.E.; Reichmuth M.L.; Fankhauser M.; Egger M.; Fenner L.	Use and caregiver-reported efficacy of medical cannabis in children and adolescents in Switzerland	European Journal of Pediatrics	2022	181	1	335-347	https://dx.doi.org/10.1007/s00431-021-04202-z	Meeting abstracts on RCTs/non-RCTs/surveys/case series or case studies with very low quality non-specific AE reporting.
Salz T.; Meza A.M.; Chino F.; Mao J.J.J.; Raghunathan N.J.; Jinna S.; Brens J.; Hernandez M.; Korenstein D.	Sociodemographic predictors of cannabis use among patients with cancer residing in states with legalized cannabis	Journal of Clinical Oncology	2022	40	28 Supplement	299	https://dx.doi.org/10.1200/JCO.2022.40.28_suppl.299	Meeting abstracts on RCTs/non-RCTs/surveys/case series or case studies with very low quality non-specific AE reporting.
Zer A.; Goshen-Lago T.G.; Ben-Aharon I.; Passhak M.	1609P Cannabis impacts on serologic status and toxic effects of the SARS-CoV-2 BNT162b2 vaccine in patients undergoing treatment for cancer	Annals of Oncology	2022	33	Supplement 7	S1278	https://dx.doi.org/10.1016/j.annonc.2022.07.1702	Meeting abstracts on RCTs/non-RCTs/surveys/case series or case studies with very low quality non-specific AE reporting.
Raghunathan N.J.; Brens J.; Vemuri S.; Mao J.J.; Korenstein D.R.	IN THE WEEDS: PATIENT INTEREST IN AND EXPERIENCE WITH CANNABIS AT A CANCER CENTER	Journal of General Internal Medicine	2022	37	Supplement 2	S152-S153	https://dx.doi.org/10.1007/s11606-022-07653-8	Meeting abstracts on RCTs/non-RCTs/surveys/case series or case studies with very low quality non-specific AE reporting.
Tofthagen C.; Starr J.; Perlman A.; Advani P.; Tan W.; Ernst B.	MEDICAL CANNABIS USE FOR CANCER RELATED SYMPTOMS AMONG FLORIDIANS: A DESCRIPTIVE STUDY	Supportive Care in Cancer	2022	30	Supplement 1	S155	https://dx.doi.org/10.1007/s00520-022-07099-y	Meeting abstracts on RCTs/non-RCTs/surveys/case series or case studies with very low quality non-specific AE reporting.
Agar M.; Naumovski V.; Chye R.; Solowij N.; Galletis P.; Liu Z.; Lintzeris N.; Currow D.; Phillips J.; Noble B.; Martin J.	Phase I/II Dose Ranging Study of the Pharmacokinetics, Dose-Response Parameters and Feasibility of Vaporised Botanical Cannabis Flower Bud in Advanced Cancer	Palliative Medicine	2022	36	1 SUPPL	71-72	https://dx.doi.org/10.1177/02692163221093145	Meeting abstracts on RCTs/non-RCTs/surveys/case series or case studies with very low quality non-specific AE reporting.
Son Y.; Fink B.; Klimowich K.; Madison I.; Scali J.; DeVincentz D.; Chialastri P.; Mueller T.; Brown G.	ANALYZING MEDICAL MARIJUANA'S EFFECT ON NARCOTIC PAIN MEDICATION CONSUMPTION IN PATIENTS WITH PROSTATE CANCER	Journal of Urology	2022	207	SUPPL 5	e458-e459	https://dx.doi.org/10.1097/JU.0000000000002570.20	Meeting abstracts on RCTs/non-RCTs/surveys/case series or case studies with very low quality non-specific AE reporting.

Authors	Title	Journal	Published Year	Volume	Issue	Pages	DOI	Exclusion Reason
Keil F.	AGMT-DISCOVER: Multicenter-randomized, double-blind-placebo-controlled, phase-III-clinical-trial to investigate effcacy + safety of Dronabinol in the Improvement of ChemOthErapy-induced and tumor-Related-symptoms in patients with locally-advanced or metas	Memo - Magazine of European Medical Oncology	2022	15	(Keil) 3rd Department of Medicine, Hematology and Oncology, Hanusch Hospital, Vienna, Austria	S59	https://dx.doi.org/10.1007/s12254-021-00793-3	Meeting abstracts on RCTs/non-RCTs/surveys/case series or case studies with very low quality non-specific AE reporting.
Grogan N.; Henry N.L.	Characterizing cannabidiol use in a breast cancer population	Cancer Research	2022	82	4 SUPPL		https://dx.doi.org/10.1158/1538-7445.SABCS21-P4-10-08	Meeting abstracts on RCTs/non-RCTs/surveys/case series or case studies with very low quality non-specific AE reporting.
Awofisayo S.O.; Eyen N.; Awofisayo J.; Arhewoh M.I.	Clinical impact: Safety and efficacy of cannabidiol "CBD" predicated on users' quality-of-life assessments in southern nigeria	International Journal of Pharmaceutical and Phytopharmacological Research	2021	11	3	21-28	https://dx.doi.org/10.51847/OTYDE20itm	No information about AEs for cancer population.
Blondin N.	Observations of complementary cannabis therapy in malignant glioma patients	Neuro-Oncology	2021	23	SUPPL 6	vi183	https://dx.doi.org/10.1093/neuonc/noab196.724	Meeting abstracts on RCTs/non-RCTs/surveys/case series or case studies with very low quality non-specific AE reporting.
Sikali J.; Fletcher C.; Gillespie A.; Hall L.; Henson J.; Hall S.; Team M.S.L.; Vitetta L.	An observational study investigating and auditing the safety, tolerability and further efficacy characteristics of a pharmaceutical grade cannabis-based medicine prescribed to eligible patients for the management of cancer related or non-cancer related pa	Asia-Pacific Journal of Clinical Oncology	2021	17	SUPPL 9	134	https://dx.doi.org/10.1111/ajco.13716	Meeting abstracts on RCTs/non-RCTs/surveys/case series or case studies with very low quality non-specific AE reporting.
Mersiades A.; Tognela A.; Kirby A.; Stockler M.; Lintzeris N.; Simes J.; Haber P.; Olver I.; Abdi E.; Della-Fiorentina S.; Fox P.; Aghmesheh M.; Chan M.; Kichendadasse G.; Briscoe K.; Wheeler H.; Sanmugarajah J.; Morton R.L.; Dieng M.; Walsh A.; Lee Y.; Grimison P.	Definitive randomised double-blind placebo-controlled trial evaluating an oral cannabinoid-rich THC/CBD cannabis extract for chemotherapy-induced nausea and vomiting (CINV): Trial in progress	Asia-Pacific Journal of Clinical Oncology	2021	17	SUPPL 9	203-204	https://dx.doi.org/10.1111/ajco.13716	Meeting abstracts on RCTs/non-RCTs/surveys/case series or case studies with very low quality non-specific AE reporting.
Gandhi A.A.G.; Khan Y.Y.K.; Nguyen B.B.N.	Patterns of use of cannabis in WA cancer patients: A survey	Asia-Pacific Journal of Clinical Oncology	2021	17	SUPPL 9	122	https://dx.doi.org/10.1111/ajco.13716	Meeting abstracts on RCTs/non-RCTs/surveys/case series or case studies with very low quality non-specific AE reporting.
Weiss M.; Danese S.; Ruiz K.A.; Kjelstrom S.; Buckley M.; Leitenberger A.; Bollmann-Jenkins M.; Larson S.; DeNittis A.S.; Martinez D.	A Survey of Breast Cancer Patients' Use of Cannabis During Radiation Therapy	International Journal of Radiation Oncology Biology Physics	2021	111	3 Supplement	e165-e166	https://dx.doi.org/10.1016/j.ijrobp.2021.07.642	Meeting abstracts on RCTs/non-RCTs/surveys/case series or case studies with very low quality non-specific AE reporting.
Bargnes V.; Mechtler L.; Ralyea C.; Hart P.; Benyovszky A.	A retrospective cohort study of medical cannabis treatment in patients with glioblastoma multiforme	Neurology	2021	96	15 SUPPL 1			Meeting abstracts on RCTs/non-RCTs/surveys/case series or case studies with very low quality non-specific AE reporting.
Bailey-Dorton C.M.; Gentile D.; Boselli D.; Yaguda S.; Greiner R.	Cannabidiol (CBD) use among cancer survivors	Journal of Clinical Oncology	2021	39	15 SUPPL		https://dx.doi.org/10.1200/JCO.2021.39.15_suppl.12096	Meeting abstracts on RCTs/non-RCTs/surveys/case series or case studies with very low quality non-specific AE reporting.
Nielsen S.; Eckhoff L.; Ruhlmann C.; Bronnum D.; Herrstedt J.; Dalton S.	Chemotherapy-induced peripheral neuropathy and cannabis use among Danish cancer patients: A cross sectional questionnaire and survey study	Supportive Care in Cancer	2021	29	SUPPL 1	S122	https://dx.doi.org/10.1007/s00520-021-06285-8	No information about AEs for cancer population.
Clarke S.J.; Vitetta L.; McLachlan A.J.; Henson J.D.; Rutolo D.; Hall S.	An oro-buccal nanoparticle delivered cannabis medicine for pain management in cancer: A clinical trial in progress	Journal of Clinical Oncology	2020	38	15		https://dx.doi.org/10.1200/JCO.2020.38.15-suppl.TPS12127	Meeting abstracts on RCTs/non-RCTs/surveys/case series or case studies with very low quality non-specific AE reporting.
Vigano A.; Canac-Marquis M.; Gamaoun R.; Beaulieu P.; Neron A.; Moride Y.; Martell M.O.; Desroches J.; Perez J.	The Quebec Cannabis Registry: a pharmacovigilance and effectiveness study on the use of medical cannabis in cancer patients	Journal of Clinical Oncology	2020	38	15		https://dx.doi.org/10.1200/JCO.2020.38.15-suppl.12109	Meeting abstracts on RCTs/non-RCTs/surveys/case series or case studies with very low quality non-specific AE reporting.
Weiss M.C.; Buckley M.; Hibbs J.; Leitenberger A.; Jenkins M.; McHugh T.W.; Green N.; Larson S.	A survey of cannabis use for symptom palliation in breast cancer patients by age and stage	Journal of Clinical Oncology	2020	38	15		https://dx.doi.org/10.1200/JCO.2020.38.15-suppl.12108	Meeting abstracts on RCTs/non-RCTs/surveys/case series or case studies with very low quality non-specific AE reporting.

Authors	Title	Journal	Published Year	Volume	Issue	Pages	DOI	Exclusion Reason
Vigano A.; Aprikian S.; Kasvis P.; Bacis V.; Al Harrasi A.; Aubin N.M.; Vigano M.; Borod M.	Safety and effectiveness of medical cannabis as a complementary option for supportive cancer care: Results from the Cannabis Pilot Project	Journal of Clinical Oncology	2020	38	15		https://dx.doi.org/10.1200/JCO.2020.38.15-suppl.12106	Meeting abstracts on RCTs/non-RCTs/surveys/case series or case studies with very low quality non-specific AE reporting.
Subbiah S.; Presant C.A.; Upadhyaya G.; Zheng M.	Cannabis use patterns and patient reported outcomes in oncology and hematology patients: An anonymous survey	Journal of Clinical Oncology	2020	38	15		https://dx.doi.org/10.1200/JCO.2020.38.15-suppl.e24143	Meeting abstracts on RCTs/non-RCTs/surveys/case series or case studies with very low quality non-specific AE reporting.
Kannappan S.; Ddamba J.; Martell K.; Arora R.; Sinha R.	EXAMINING CANNABIS USAGE AND CANNABIS-RELATED INFORMATION SOURCING FOR DISEASE MANAGEMENT IN CLINICAL ONCOLOGY	Radiotherapy and Oncology	2020	150	Supplement 1	S23	https://dx.doi.org/10.1016/S0167-8140%2820%2930938-5	Meeting abstracts on RCTs/non-RCTs/surveys/case series or case studies with very low quality non-specific AE reporting.
Shi R.	Topical cannabis-based medicines: A new treatment for integumentary neoplasms	Journal of Wound Care	2020	29	SUPPL 7B	76		Meeting abstracts on RCTs/non-RCTs/surveys/case series or case studies with very low quality non-specific AE reporting.
Chan M.M.; Roberts-Thomson R.	The current practice and understanding of medical cannabis use in a single institution medical oncology outpatient setting	Asia-Pacific Journal of Clinical Oncology	2020	16	SUPPL 2	31	https://dx.doi.org/10.1111/ajco.13417	Meeting abstracts on RCTs/non-RCTs/surveys/case series or case studies with very low quality non-specific AE reporting.
Velazquez Ramos P.M.; Galzerano J.	Medical cannabis as a therapeutic resource: Preliminary study	Medical Cannabis and Cannabinoids	2020	3	2	129	https://dx.doi.org/10.1159/000505827	No information about AEs for cancer population.
Ibrahim M.E.	Cannabis between medical uses and recreational uses	Medical Cannabis and Cannabinoids	2020	3	2	118-119	https://dx.doi.org/10.1159/000505827	No information about AEs for cancer population.
Clarke S.; Butcher B.; McLachlan A.J.; Henson J.D.; Rutolo D.; Hall S.; Vitetta L.	Pilot clinical and pharmacokinetic study of a water soluble nanoparticle cannabis-based medicine in advanced cancer with intractable pain	Asia-Pacific Journal of Clinical Oncology	2020	16	SUPPL 8	117-118	https://dx.doi.org/10.1111/ajco.13498	Meeting abstracts on RCTs/non-RCTs/surveys/case series or case studies with very low quality non-specific AE reporting.
Grimison P.S.; Stockler M.R.; Kirby A.; Walsh A.; Lintzeris N.; Cheung Y.; Mersiades A.; Tognela A.; Haber P.; Simes J.; Morton R.L.; Olver I.N.; McGregor I.; Gedye C.; Fox P.; Briscoe K.P.; Aghmesheh M.; Abdi E.A.; Della-Fiorentina S.	Results of crossover phase 2 component of randomised placebo-controlled trial evaluating oral THC/CBD cannabis extract for refractory chemotherapy-induced nausea and vomiting (CINV)	Asia-Pacific Journal of Clinical Oncology	2020	16	SUPPL 8	206-207	https://dx.doi.org/10.1111/ajco.13498	Meeting abstracts on RCTs/non-RCTs/surveys/case series or case studies with very low quality non-specific AE reporting.
Kirkova Y.; Hughes C.	Care of the elderly cognitively impaired palliative patient with cancer	Journal of the American Geriatrics Society	2020	68	SUPPL 1	S195-S196	https://dx.doi.org/10.1111/jgs.16431	Meeting abstracts on RCTs/non-RCTs/surveys/case series or case studies with very low quality non-specific AE reporting.
Webster E.M.; Yadav G.; Gysler S.; McNamara B.; Black J.D.; Tymon-Rosario J.R.; Zeybek B.; Han C.; Menderes G.; Huang G.S.; Silasi D.A.; Azodi M.; Schwartz P.E.; Santin A.D.; Ratner E.S.; Altwerger G.	Patient experience with medical marijuana in women with gynecologic malignancies: A single-institution survey-based study	Gynecologic Oncology	2020	159	Supplement 1	318	https://dx.doi.org/10.1016/j.ygyno.2020.05.575	Meeting abstracts on RCTs/non-RCTs/surveys/case series or case studies with very low quality non-specific AE reporting.
Aubrey R.; Chun H.; Feuz C.; Rosewall T.	Cannabis Use by Cancer Patients: A Thematic Analysis of Patient-Initiated Cancer Blog Posts	Journal of Medical Imaging and Radiation Sciences	2020	51	3 Supplement	S17	https://dx.doi.org/10.1016/j.jmir.2020.07.050	Meeting abstracts on RCTs/non-RCTs/surveys/case series or case studies with very low quality non-specific AE reporting.
Lacey J.; Schloss J.M.; Sinclair J.; Steel A.; Sughrue M.; Teo C.; Sibbritt D.	A phase II double-blind, randomized clinical trial assessing the tolerability of two different ratios of cannabis in patients with glioblastoma multiforme (GBM)	Journal of Clinical Oncology	2020	38	15		https://dx.doi.org/10.1200/JCO.2020.38.15_suppl.2530	Meeting abstracts on RCTs/non-RCTs/surveys/case series or case studies with very low quality non-specific AE reporting.
Battle D.; Bergerot C.D.; Msaouel P.; Zhang T.; George D.J.; Staehler M.D.	Patient-reported use of marijuana and cannabinoid (CBD) oil in patients with renal cell carcinoma undergoing systemic therapy	Journal of Clinical Oncology	2020	38	15		https://dx.doi.org/10.1200/JCO.2020.38.15_suppl.5084	Meeting abstracts on RCTs/non-RCTs/surveys/case series or case studies with very low quality non-specific AE reporting.
Weiss M.C.; Hibbs J.; McHugh T.W.; Buckley M.; Larson S.; Green N.; Kaklamani V.G.; Chlebowski R.T.; Martinez D.	A survey of breast cancer patients' use of cannabis before, during, and after treatment	Journal of Clinical Oncology	2020	38	15		https://dx.doi.org/10.1200/JCO.2020.38.15_suppl.e19210	Meeting abstracts on RCTs/non-RCTs/surveys/case series or case studies with very low quality non-specific AE reporting.
Benkli B.; Ansoanuur G.; Bonfante Mejia E.; Smart S.	Levetiracetam in management of bilateral trigeminal neuralgia due to large glomus tumor	Headache	2020	60	Supplement 1	42-43	https://dx.doi.org/10.1111/head.13854	Meeting abstracts on RCTs/non-RCTs/surveys/case series or case studies with very low quality non-specific AE reporting.

Authors	Title	Journal	Published Year	Volume	Issue	Pages	DOI	Exclusion Reason
Mechtler L.; Ralyea C.; Hart P.; Bargnes V.	The use of Medical Cannabis in the Treatment of Neuropathies: An Ongoing Retrospective Study	European Journal of Neurology	2020	27	Supplement 1	863		Meeting abstracts on RCTs/non-RCTs/surveys/case series or case studies with very low quality non-specific AE reporting.
Battle D.; Rathmell W.K.; Bergerot C.; Msaouel P.; Jonasch E.; George D.; Zhang T.; Staehler M.	Underreporting of side effects in systemic therapy for renal cell carcinoma	Journal of Urology	2020	203	Supplement 4	e202		Meeting abstracts on RCTs/non-RCTs/surveys/case series or case studies with very low quality non-specific AE reporting.
Dolan A.; Portman D.; Donovan K.	Reasons for discontinuing marijuana use in young adult (YA) cancer patients	Psycho-Oncology	2020	29	Supplement 1	64-65	https://dx.doi.org/10.1002/pon.5327	Meeting abstracts on RCTs/non-RCTs/surveys/case series or case studies with very low quality non-specific AE reporting.
Dolan A.; Portman D.; Donovan K.	Young adult cancer patients and marijuana: Characteristics and patterns of use	Psycho-Oncology	2020	29	Supplement 1	63	https://dx.doi.org/10.1002/pon.5327	Meeting abstracts on RCTs/non-RCTs/surveys/case series or case studies with very low quality non-specific AE reporting.
Mousa A.; Petrovic M.; Fleshner N.E.	Prevalence and predictors of cannabis use among men receiving androgen-deprivation therapy for advanced prostate cancer	Canadian Urological Association Journal	2019	14	1	E20-E26	https://dx.doi.org/10.5489/CUAJ.5911	No information about AEs for cancer population.
do Nascimento R.G.F.O.; de Souza L.G.; Vieira Santos F.R.P.; de Souza Andrade Filho A.	Cannabinoides use in the control of convulsive crisis in astrocytoma after neurosurgery	Revista Brasileira de Neurologia e Psiquiatria	2019	23	2	177-186		Non-English language article.
Likar R.; Koestenberger M.; Stultschnig M.; Nahler G.	Concomitant treatment of malignant brain tumours with CBD - A case series and review of the literature	Anticancer Research	2019	39	10	5797-5801	https://dx.doi.org/10.21873/anticanres.13783	No information about AEs for cancer population.
Chang Y.D.; Jung J.-W.; Oberoi-Jassal R.; Kim J.; Rajasekhara S.; Haas M.; Smith J.; Desai V.; Donovan K.A.; Portman D.	Edmonton symptom assessment scale and clinical characteristics associated with cannabinoid use in oncology supportive care outpatients	JNCCN Journal of the National Comprehensive Cancer Network	2019	17	9	1059-1064	https://dx.doi.org/10.6004/jnccn.2019.7301	No information about AEs for cancer population.
Ueberall M.A.; Essner U.; Mueller-Schwefe G.H.H.	Effectiveness and tolerability of THC:CBD oromucosal spray as add-on measure in patients with severe chronic pain: Analysis of 12-week open-label real-world data provided by the German pain e-registry	Journal of Pain Research	2019	12	(Ueberall) Institute of Neurological Sciences, Nuernberg 90411, Germany(Essner) O.Meany Consultancy, Hamburg 22339, Germany(Mueller-Schwefe) Interdisciplinary Centre for Pain and Palliative Care Medicine, Goeppingen 73033, Germany	1577-1604	https://dx.doi.org/10.2147/JPR.S192174	No information about AEs for cancer population.
Melen C.M.; Merrien M.; Wasik A.; Sonnevi K.; Junlen H.-R.; Christersson B.; Sander B.; Wahlin B.E.	A Clinical Trial of Cannabis As Targeted Therapy for Indolent Leukemic Lymphoma	Blood	2019	134	Supplement 1	5487	https://dx.doi.org/10.1182/blood-2019-126693	Meeting abstracts on RCTs/non-RCTs/surveys/case series or case studies with very low quality non-specific AE reporting.
Slaven M.; Levine M.; Parpia S.; Shaw E.	An approach to dosing: The cannabis oil in pain effectiveness (COPE) trial	Medical Cannabis and Cannabinoids	2019	2	2	2	https://dx.doi.org/10.1159/000502323	Meeting abstracts on RCTs/non-RCTs/surveys/case series or case studies with very low quality non-specific AE reporting.
Dusi V.; Attili S.V.S.; Singaraju M.	Observational study on role of crude cannabis in pain control and quality of life in terminally ill cancer patients: An Indian perspective	Annals of Oncology	2019	30	Supplement 9	ix119	https://dx.doi.org/10.1093/annonc/mdz430.005	Meeting abstracts on RCTs/non-RCTs/surveys/case series or case studies with very low quality non-specific AE reporting.
Stern K.M.; Godbole R.; Ejadi S.; Gallegos N.; Benn B.	What CAM it be? a case of metastatic pancreatic adenocarcinoma confounded by complementary and alternative medicine (CAM) therapies	American Journal of Respiratory and Critical Care Medicine	2019	199	9			Meeting abstracts on RCTs/non-RCTs/surveys/case series or case studies with very low quality non-specific AE reporting.

Authors	Title	Journal	Published Year	Volume	Issue	Pages	DOI	Exclusion Reason
Macari D.; Gbadamosi B.; Ezekwudo D.; Khoury J.; Jaiyesimi I.A.; Gaikazian S.S.	Medical cannabis in cancer patients: Prevalence, efficacy, and safety	Journal of Clinical Oncology	2019	37	Supplement 15		https://dx.doi.org/10.1200/JCO.2019.37.15_suppl.e23099	Meeting abstracts on RCTs/non-RCTs/surveys/case series or case studies with very low quality non-specific AE reporting.
Gamaoun R.; Kasvis P.; Patronidis F.; Arboleda M.F.; Vigano A.	Potential impact of medical cannabis treatment on common symptoms improvement using the edmonton symptom assessment scale among cancer patients in Quebec-Canada: Pilot study	Supportive Care in Cancer	2019	27	1 Supplement	S80-S81	https://dx.doi.org/10.1007/s00520-019-04813-1	Meeting abstracts on RCTs/non-RCTs/surveys/case series or case studies with very low quality non-specific AE reporting.
Brodsky A.L.; Gerber D.; Lutz K.; Reese E.; Pothuri B.; Kim A.	Medical marijuana for palliation of symptoms in women with gynecologic cancers	Gynecologic Oncology	2019	154	Supplement 1	161-162	https://dx.doi.org/10.1016/j.ygyno.2019.04.380	Meeting abstracts on RCTs/non-RCTs/surveys/case series or case studies with very low quality non-specific AE reporting.
Singh V.; Zarrabi A.J.; Welsh J.; Gillespie T.W.; Curseen K.A.; Baer W.; McKenzie-Brown A.M.; Sniecinski R.M.	General beliefs/perspectives of patients with low-THC oil card regarding marijuana-related products	Pain Medicine (United States)_1	2019	20	3	659	https://dx.doi.org/10.1093/pm/pny317	Meeting abstracts on RCTs/non-RCTs/surveys/case series or case studies with very low quality non-specific AE reporting.
Wilson M.; Masterson E.; Broglio K.	Cannabis Use Among Patients Prescribed Opioids in a Palliative Care Clinic (S875)	Journal of Pain and Symptom Management	2019	57	2	522	https://dx.doi.org/10.1016/j.jpainsymman.2018.12.325	Meeting abstracts on RCTs/non-RCTs/surveys/case series or case studies with very low quality non-specific AE reporting.
Arboleda M.F.; Chamberland Prosk E.G.; Vigano A.	Medical cannabis oil: Results from the first-in-human phase 1, double-blind, randomized, placebo-controlled study	Medical Cannabis and Cannabinoids	2018	1	2	123	https://dx.doi.org/10.1159/000493905	No information about AEs for cancer population.
Blondin N.	The evolving role of complementary cannabis therapy in glioblastoma treatment	Neuro-Oncology	2018	20	Supplement 6	vi214-vi215	https://dx.doi.org/10.1093/neuonc/noy148	Meeting abstracts on RCTs/non-RCTs/surveys/case series or case studies with very low quality non-specific AE reporting.
Aggarwal S.; Bhowmick J.; Sharma R.; Singh M.; Gond R.K.; Dash I.; Aggarwal A.	Voice of cancer patients (VoCP): Patient perceptions regarding use of marijuana and its derivatives in cancer	Journal of Clinical Oncology	2018	36	34 Supplement		https://dx.doi.org/10.1200/JCO.2018.36.34_suppl.228	Meeting abstracts on RCTs/non-RCTs/surveys/case series or case studies with very low quality non-specific AE reporting.
Bulbul A.; Mino E.A.; Khorsand-Sahbaie M.; Lentkowski L.	Opioid dose reduction and pain control with medical cannabis	Journal of Clinical Oncology	2018	36	34 Supplement		https://dx.doi.org/10.1200/JCO.2018.36.34_suppl.189	Meeting abstracts on RCTs/non-RCTs/surveys/case series or case studies with very low quality non-specific AE reporting.
Temple L.M.; Lampert S.L.	Descriptive study of medical cannabis patients in the Illinois compassionate use of medical cannabis pilot program: Opportunities for improving patient care	Global Advances in Health and Medicine	2018	7	(Temple) Northshore University Health System, Glenview, IL, United States(Lampert) Lehigh University, Glencoe, IL, United States	93	https://dx.doi.org/10.1177/2164956118773837	Meeting abstracts on RCTs/non-RCTs/surveys/case series or case studies with very low quality non-specific AE reporting.
Arboleda M.-F.; Dam V.; Prosk E.; Dworkind M.; Vigano A.	Cannabis-Based Medications: The Future Co-analgesics of Choice for Cancer Patients?	Journal of Pain and Symptom Management	2018	56	6	e68	https://dx.doi.org/10.1016/j.jpainsymman.2018.10.191	Meeting abstracts on RCTs/non-RCTs/surveys/case series or case studies with very low quality non-specific AE reporting.
Dini E.; Cafalli M.; De Luca C.; Baldacci F.; Gori S.; Bonuccelli U.	Case report: Chronic migraine successfully treated with cannabinoids	Journal of Headache and Pain	2018	19	Supplement 1		https://dx.doi.org/10.1186/s10194-018-0900-0	No information about AEs for cancer population.
Van Den Hengel-Koot I.S.; Nugteren-Van Lonkhuyzen J.J.; Hondebrink L.; Rietjens S.J.; De Vries I.; Van Riel A.J.H.P.	Unexpected toxicity of cannabidiol (CBD) oil health products	Clinical Toxicology	2018	56	6	516	https://dx.doi.org/10.1080/15563650.2018.1457818	No information about AEs for cancer population.
Mousa A.; Petrovic M.; Laszlo S.; Fleshner N.	Is there a therapeutic role for cannabis in advanced prostate cancer? Exploring the patterns and predictors of use among men receiving androgen-deprivation therapy	Canadian Urological Association Journal	2018	12	6 Supplement 2	S126		Meeting abstracts on RCTs/non-RCTs/surveys/case series or case studies with very low quality non-specific AE reporting.
Bar-Sela G.; Tauber D.; Mitnik I.; Sheinman-Yuffe H.; Aharon-Peretz J.	Cannabis-related cognitive impairment: Prospective evaluation of possible influences on cancer patients during chemotherapy treatment	Supportive Care in Cancer	2018	26	2 Supplement 1	S156	https://dx.doi.org/10.1007/s00520-018-4193-2	Meeting abstracts on RCTs/non-RCTs/surveys/case series or case studies with very low quality non-specific AE reporting.
Vigano M.; Arboleda M.F.; Prosk E.; Drozd Y.; Xuecheng L.; Dworkind M.	Cannabis-based medicines showmore promise for improving appetite in chronic diseases	Supportive Care in Cancer	2018	26	2 Supplement 1	S54	https://dx.doi.org/10.1007/s00520-018-4193-2	Meeting abstracts on RCTs/non-RCTs/surveys/case series or case studies with very low quality non-specific AE reporting.

Authors	Title	Journal	Published Year	Volume	Issue	Pages	DOI	Exclusion Reason
Arboleda M.F.; Dam V.; Drozd Y.; Prosk E.; Kasvis P.; Dworkind M.; Kilgour R.; Vigano A.	Effect of medical cannabis on appetite and weight: A retrospective analysis	Journal of Cachexia, Sarcopenia and Muscle	2018	9	1	205-206	https://dx.doi.org/10.1002/jcsm.12284	Meeting abstracts on RCTs/non-RCTs/surveys/case series or case studies with very low quality non-specific AE reporting.
Polito S.; Dupuis L.L.; Sung L.; Patel P.; Ning W.; Khanna M.	Nabilone for prevention of acute chemotherapy-induced nausea and vomiting in children: A single centre retrospective review	Canadian Journal of Hospital Pharmacy	2017	70	1	67		Meeting abstracts on RCTs/non-RCTs/surveys/case series or case studies with very low quality non-specific AE reporting.
Hansra D.; Granada H.	Evaluation of safety, efficacy, and clinical endpoints of delta-9-tetrahydrocannabinol in patients age 60 or older with hematologic and oncologic malignancies	Blood	2017	130	Supplement 1			Meeting abstracts on RCTs/non-RCTs/surveys/case series or case studies with very low quality non-specific AE reporting.
Short S.C.; Little C.	A 2-part safety and exploratory efficacy randomised double-blind, placebo-controlled study of a 1:1 ratio of cannabidiol and delta-9-tetrahydrocannabinol (CBD: THC) plus doseintense temozolomide in patients with recurrent glioblastoma multiforme (GBM)	Neuro-Oncology	2017	19	Supplement 6	vi13	https://dx.doi.org/10.1093/neuonc/nox168	Meeting abstracts on RCTs/non-RCTs/surveys/case series or case studies with very low quality non-specific AE reporting.
Taha T.; Talhamy S.; Wollner M.; Peer A.; Bar-Sela G.	The effect of cannabis use on tumor response to nivolumab in patients with advanced malignancies	Annals of Oncology	2017	28	Supplement 5	v544		Meeting abstracts on RCTs/non-RCTs/surveys/case series or case studies with very low quality non-specific AE reporting.
Lacey J.	Prescribing medicinal cannabis for chemotherapy induced nausea and vomiting and refractory advanced cancer related symptoms: A prescribing physician's perspective	Asia-Pacific Journal of Clinical Oncology	2017	13	Supplement 4	216	https://dx.doi.org/10.1111/ajco.12799	Meeting abstracts on RCTs/non-RCTs/surveys/case series or case studies with very low quality non-specific AE reporting.
Menniti-Ippolito F.; Da Cas R.; Gallo E.; Firenzuoli F.	Suspected adverse reactions to cannabis galenic preparations for medical use in Italy	Drug Safety	2017	40	10	964-965	https://dx.doi.org/10.1007/s40264-017-0580-8	Meeting abstracts on RCTs/non-RCTs/surveys/case series or case studies with very low quality non-specific AE reporting.
Hansra D.M.	Evaluation of safety, efficacy, and other clinical endpoints of delta-9-tetrahydrocannabinol in older patients with hem/onc malignancies	Journal of Clinical Oncology	2017	35	15 Supplement 1			Meeting abstracts on RCTs/non-RCTs/surveys/case series or case studies with very low quality non-specific AE reporting.
Twelves C.; Short S.; Wright S.	A two-part safety and exploratory efficacy randomized double-blind, placebo-controlled study of a 1:1 ratio of the cannabinoids cannabidiol and delta-9-tetrahydrocannabinol (CBD: THC) plus dose-intense temozolomide in patients with recurrent glioblastoma	Journal of Clinical Oncology	2017	35	15 Supplement 1			Meeting abstracts on RCTs/non-RCTs/surveys/case series or case studies with very low quality non-specific AE reporting.
Krpina K.; Jakopovic M.; Roglic M.	Use of alternative therapy in patients with lung cancer	Journal of Thoracic Oncology	2017	12	1 Supplement 1	S608-S609		Meeting abstracts on RCTs/non-RCTs/surveys/case series or case studies with very low quality non-specific AE reporting.
Sweeney B.; Talebi S.; Toro D.; Gonzalez K.; Menoscal J.-P.; Shaw R.; Hassen G.W.	Hyperthermia and severe rhabdomyolysis from synthetic cannabinoids	American Journal of Emergency Medicine	2016	34	1	e1-121	https://dx.doi.org/10.1016/j.ajem.2015.05.052	No information about AEs for cancer population.
Luckett T.; Agar M.; Chye R.; Lintzeris N.; McGregor I.; Allsop D.; Noble B.; Clark K.; Lovell M.; Lee J.; Martin P.; Sheehan C.; Aggarwal R.; Pene C.; Solowij N.; Martin J.; Devilee L.; Currow D.; Phillips J.	Medicinal cannabis use and preferred mode of administration: Preliminary results from an anonymous patient survey to inform medicinal cannabis phase II and III trials for cancer-related anorexia-cachexia	Palliative Medicine	2016	30	6	NP88	https://dx.doi.org/10.1177/0269216316646056	Meeting abstracts on RCTs/non-RCTs/surveys/case series or case studies with very low quality non-specific AE reporting.
Juarez T.; Piccioni D.; Nguyen A.; Brown B.; Rose L.; Pu M.; Messer K.; Kesari S.	A phase I dose escalation and central nervous system (CNS) pharmacokinetic study of dexanabinol in patients with brain cancer	Neuro-Oncology	2015	17	SUPPL. 5	v13	https://dx.doi.org/10.1093/neuonc/nov205.13	Meeting abstracts on RCTs/non-RCTs/surveys/case series or case studies with very low quality non-specific AE reporting.
Plummer R.; Anthoney A.; Evans J.; Haris N.; D'Archangelo M.; Slater S.; Campbell S.; Brindley C.; Self S.; McKeown P.; Lavin S.; Hynes D.; Flores M.V.; Pedret-Dunn A.; Laffranchi B.	A phase I dose escalation study to assess the safety tolerability and pharmacokinetics of ETS2101 in patients (pts) with advanced solid tumours	European Journal of Cancer	2015	51	SUPPL. 3	S58-S59		Meeting abstracts on RCTs/non-RCTs/surveys/case series or case studies with very low quality non-specific AE reporting.
Brunt T.M.; Van Genugten M.; Honer-Snoeken K.; Van De Velde M.J.; Niesink R.J.M.	Therapeutic satisfaction and subjective effects of different strains of pharmaceutical-grade cannabis	Journal of Clinical Psychopharmacology	2014	34	3	344-349	https://dx.doi.org/10.1097/JCP.0000000000000129	No information about AEs for cancer population.

Authors	Title	Journal	Published Year	Volume	Issue	Pages	DOI	Exclusion Reason
Notcutt W.; Phillips C.; Hughes J.; Lacoux P.; Vijayakulasingam V.; Baldock L.	A retrospective description of the use of nabilone in UK clinical practice	Multiple Sclerosis	2014	20	1 SUPPL. 1	468	https://dx.doi.org/10.1177/1352458514547846	Meeting abstracts on RCTs/non-RCTs/surveys/case series or case studies with very low quality non-specific AE reporting.
Elder J.J.; Knoderer H.M.	Characterization of cannabinoid usage in a pediatric oncology population	Pharmacotherapy	2011	31	10	369e		Meeting abstracts on RCTs/non-RCTs/surveys/case series or case studies with very low quality non-specific AE reporting.
Radiano R.; Or R.; Shouval R.; Mechoulam R.; Abu-Zaid B.	Improved symptom management by use of medical cannabis	Bone Marrow Transplantation	2011	46	SUPPL. 1	S394-S395	https://dx.doi.org/10.1038/bmt.2011.50	Meeting abstracts on RCTs/non-RCTs/surveys/case series or case studies with very low quality non-specific AE reporting.
Koula-Jenik H.; Holzhauer P.	Observational study for preventive potential of hempseed oil in capecitabine-induced hand-foot syndrome	Deutsche Zeitschrift fur Onkologie	2010	42	2	80-84	https://dx.doi.org/10.1055/s-0029-1242613	Non-English language article.
Gingerich J.; Wadhwa D.; Lemanski L.; Krahn M.; Daeninck P.J.	The use of cannabinoids (CBs) for the treatment of chemotherapy-induced peripheral neuropathy (CIPN): A retrospective review	Journal of Clinical Oncology	2009	27	15 SUPPL. 1	e20743		Meeting abstracts on RCTs/non-RCTs/surveys/case series or case studies with very low quality non-specific AE reporting.
Lynch M.E.; Young J.; Clark A.J.	A Case Series of Patients Using Medicinal Marihuana for Management of Chronic Pain Under the Canadian Marihuana Medical Access Regulations	Journal of Pain and Symptom Management	2006	32	5	497-501	https://dx.doi.org/10.1016/j.jpainsymman.2006.05.016	No information about AEs for cancer population.
Ware M.A.; Adams H.; Guy G.W.	The medicinal use of cannabis in the UK: Results of a nationwide survey	International Journal of Clinical Practice	2005	59	3	291-295	https://dx.doi.org/10.1111/j.1742-1241.2004.00271.x	No information about AEs for cancer population.
Grotenhermen F.; Schnelle M.	Survey on the medical use of Cannabis and THC in Germany	Journal of Cannabis Therapeutics	2003	3	2	17-40	https://dx.doi.org/10.1300/J175v03n02_03	No information about AEs for cancer population.
Gallagher R.; Best J.A.; Fyles G.; Hawley P.; Yeomans W.	Attitudes and beliefs about the use of Cannabis for symptom control in a palliative population	Journal of Cannabis Therapeutics	2003	3	2	41-50	https://dx.doi.org/10.1300/J175v03n02_04	No information about AEs for cancer population.
Perez E.A.; Lembersky B.; Kaywin P.; Kalman L.; Yocom K.; Friedman C.	Comparable safety and antiemetic efficacy of a brief (30-second bolus) intravenous granisetron infusion and a standard (15-minute) intravenous ondansetron infusion in breast cancer patients receiving moderately emetogenic chemotherapy	Cancer Journal from Scientific American	1998	4	1	52-58		Wrong intervention (not CBP).
Higi M.; Niederle N.; Bremer K.; Schmitt G.; Schmidt C.G.; Seeber S.	Levonantradol in the treatment of nausea and vomiting caused by cytostatic drugs	Deutsche Medizinische Wochenschrift	1982	107	33	1232-1234	https://dx.doi.org/10.1055/s-2008-1070107	Non-English language article.
George M.; Pejovic M.H.; Thuaire M.	Randomized trial of nabilone as antimetic in cancer patients treated with cisplatin	Biomedicine and Pharmacotherapy	1983	37	1	24-27		Non-English language article.
Heim M.E.; Queisser W.	Treatment of refractory cancer chemotherapy induces vomiting with the synthetic cannabinoid levonantradol	Onkologie	1982	5	2	94-96		Non-English language article.
Broder L.E.; Lean N.L.; Hilsenbeck S.G.	A randomized blinded clinical trial comparing delta-9-tetrahydrocannabinol (THC) and hydroxizine (HZ) as antiemetics (AE) for cancer chemotherapy (CT)	Proceedings of the American Association for Cancer Research	1982	Vol. 23	(Broder, Lean, Hilsenbeck) Comprehensive Cancer Cent. State Florida, Univ. Miami Sch. Med., Miami, FL 33136 United States	514		Meeting abstracts on RCTs/non-RCTs/surveys/case series or case studies with very low quality non-specific AE reporting.
Colls B.M.	Cytotoxic nausea and cannabinoids	Lancet	1981	1	8224	833		Hearsay/opinion on AEs.
Kluin-Nelemans J.C.; Meuwissen Th. O.J.A.; Nelemans F.A.; Maes R.A.A.	DELTA9-Tetrahydrocannabinol (THC) as an anti-emetic in patients treated with cancer chemotherapy. A double-blind cross-over trial against placebo	Netherlands Journal of Medicine	1981	24	2	90		Meeting abstracts on RCTs/non-RCTs/surveys/case series or case studies with very low quality non-specific AE reporting.

Authors	Title	Journal	Published Year	Volume	Issue	Pages	DOI	Exclusion Reason
Riggs Jr C.E.; Duffey P.L.; Egorin M.J.; Bachur N.R.	Influence of delta-9-tetrahydrocannabinol on metabolism and plasma elimination of adriamycin and cyclophosphamide	Proceedings of the American Association for Cancer Research	1981	Vol. 22	(Riggs Jr, Duffey, Egorin, Bachur) Lab. Clin. Biochem., Clin. Oncol. Branch, Baltimore Canc. Res. Program, DCT, NCI, NIH, Baltimore, Md. 21201 United States	C-79		Meeting abstracts on RCTs/non-RCTs/surveys/case series or case studies with very low quality non-specific AE reporting.
Kluin-Nelemans J.C.; Meuwissen Th. O.J.A.; Nelemans F.A.; Maes R.A.A.	Tetrahydrocannabinol as antiemetic in patients treated with cytostatics: double blind crossover trial against placebo	Nederlands Tijdschrift voor Geneeskunde	1981	125	22	900-901		Non-English language article.
Colls B.M.	Cannabis and cancer chemotherapy	Lancet	1980	1	8179	1187-1188		Meeting abstracts on RCTs/non-RCTs/surveys/case series or case studies with very low quality non-specific AE reporting.
Chang A.E.; Shiling D.J.; Stillman R.C.	A prospective randomized trial of delta-9-tetrahydrocannabinol (THC) as an antiemetic in patients receiving high dose methotrexate (MTX)	Proceedings of the American Association for Cancer Research	1979	Vol. 20	(Chang, Shiling, Stillman) Dept. Surg. Branch, Nat. Cancer Inst., Bethesda, Md. 20014 United States	No.-357		Meeting abstracts on RCTs/non-RCTs/surveys/case series or case studies with very low quality non-specific AE reporting.
Frytak S.; Moertel C.G.; O'Fallon J.R.	A comparison of delta-9-tetrahydrocannabinol (THC), prochlorperazine (PCP) and placebo as antiemetics for cancer chemotherapy	Proceedings of the American Association for Cancer Research	1979	Vol. 20	(Frytak, Moertel, O'Fallon) Mayo Clin., Rochester, Minn. 55901 United States	No.C-414		Meeting abstracts on RCTs/non-RCTs/surveys/case series or case studies with very low quality non-specific AE reporting.
Sallan S.; Zinberg N.; Frei E.	Oral delta 9 tetrahydrocannabinol (THC) in the prevention of vomiting (V) associated with cancer chemotherapy (CC)	Proceedings of the American Association for Cancer Research	1975	16	66	No.-575		Meeting abstracts on RCTs/non-RCTs/surveys/case series or case studies with very low quality non-specific AE reporting.
Kluin-Nelemans, J.C.; Meuwissen Th., O.J.A.; Nelemans, F.A.; Maes, R.A.A.	Î”9-Tetrahydrocannabinol (THC) as an anti-emetic in patients treated with cancer chemotherapy. A double-blind cross-over trial against placebo	NETH. J. MED.	1981	24	2	90		Meeting abstracts on RCTs/non-RCTs/surveys/case series or case studies with very low quality non-specific AE reporting.
WONGKONGDECH, Ranee; PANSILA, Narisara; NONETOOM, Pichit; TURNBULL, Niruwan; WONGKONGDECH, Adisorn	Symptom Management and Quality of Life of Palliative Cancer Patients After Being Administered with Thai Medicinal Cannabis ...International Conference on Informatics, Management, and Technology in Healthcare (ICIMTH), 1-3 July, 2022, Athens, Greece.	Studies in Health Technology & Informatics	2022	295		450-453	10.3233/SHTI220762	No information about AEs for cancer population.
Wolfe, Joanne; Bluebond-Langner, Myra; Doherty, Megan; Power, Liam; Attala, Monica; Vadeboncoeur, Christina	Use of oral cannabis extracts in the pediatric palliative care setting: A retrospective chart review.	Palliative Medicine	2020	34	3	435-437	10.1177/0269216320904315	No information about AEs for cancer population.
RICHARDSON, PAMELA	The Case of the Cannabis Conundrum.	ONS Connect	2014	29	4	11-Nov		No information about AEs for cancer population.
	Clinical digest. Marijuana use linked to the worst prognoses of testicular cancer.	Nursing Standard	2012	27	6	15-15	10.7748/ns.27.6.15.s23	Wrong concept (study reporting/evaluating the risk of developing cancer associated with CBP use in other populations).
Silman, YS; Eshet, L; Kennett, R; ben Ami, S	THE EFFECT OF CANNABIS CONSUMPTION ON IMMUNE RELATED ADVERSE EVENTS IN MELANOMA PATIENTS TREATED WITH IPILIMUMAB NIVOLUMAB COMBINATION THERAPY	ONCOLOGY NURSING FORUM	2021	48	2			Meeting abstracts on RCTs/non-RCTs/surveys/case series or case studies with very low quality non-specific AE reporting.

S5. Excluded Articles

Authors	Title	Journal	Published Year	Volume	Issue	Pages	DOI	Exclusion Reason
Stith, SS; Vigil, JM; Brockelman, F; Keeling, K; Hall, B	The Association between Cannabis Product Characteristics and Symptom Relief	SCIENTIFIC REPORTS	2019	9			10.1038/s41598-019-39462-1	No information about AEs for cancer population.
Guide, JG; Navone, CCO; Perez, MDR; Gonzalez, ALC; Ramos, PMV	Medical cannabis as a therapeutic resource: preliminary study	REVISTA MEDICA DEL URUGUAY	2019	35	4	289-297	10.29193/RMU.35.4.5	Non-English language article.
Gschiel, B; Likar, R	Cannabinoids in Palliative Symptom Control	ZEITSCHRIFT FUR PALLIATIVMEDIZIN	2009	10	1	45-50	10.1055/s-0028-1090169	Non-English language article.
Likar, R; Pipam, W; Kostenberger, M; Neuwersch, S	Cannabinoids in Clinical Practice Dronabinol for the Treatment of Therapy-Resistant Nausea and Vomiting of Cancer Patients in Palliative Care	ZEITSCHRIFT FUR PALLIATIVMEDIZIN	2017	18	5	249-254	10.1055/s-0043-109511	Non-English language article.
Lutoti, S; Kaggwa, B; Kamba, PF; Mukonzo, J; Sesaazi, CD; Katuura, E	Ethnobotanical Survey of Medicinal Plants Used in Breast Cancer Treatment by Traditional Health Practitioners in Central Uganda	JOURNAL OF MULTIDISCIPLINARY HEALTHCARE	2023	16		635-651	10.2147/JMDH.S387256	No information about AEs for cancer population.
Stith, SS; Li, XX; Orozco, J; Lopez, V; Brockelman, F; Keeling, K; Hall, B; Vigil, JM	The Effectiveness of Common Cannabis Products for Treatment of Nausea	JOURNAL OF CLINICAL GASTROENTEROLOGY	2022	56	4	331-338	10.1097/MCG.0000000000001534	No information about AEs for cancer population.
Casarett, DJ; Beliveau, JN; Arbus, MS	Benefit of Tetrahydrocannabinol versus Cannabidiol for Common Palliative Care Symptoms	JOURNAL OF PALLIATIVE MEDICINE	2019	22	10	1180-1184	10.1089/jpm.2018.0658	No information about AEs for cancer population.
Butler, TW; Hande, K; Ryan, M; Raman, R; McDowell, MR; Cones, B; Jackson, HJ; Cortez, M; Murphy, BA	Cannabidiol Knowledge, beliefs, and experiences of patients with cancer	CLINICAL JOURNAL OF ONCOLOGY NURSING	2021	25	4	405-412	10.1188/21.CJON.405-412	No information about AEs for cancer population.
Kaar, SJ; Gao, CX; Lloyd, B; Smith, K; Lubman, DI	Trends in cannabis-related ambulance presentations from 2000 to 2013 in Melbourne, Australia	DRUG AND ALCOHOL DEPENDENCE	2015	155		24-30	10.1016/j.drugalcdep.2015.08.021	No information about AEs for cancer population.
