# Supplementary material for: Adverse events associated with the use of cannabis-based products in people living with cancer: a systematic scoping review
Source: Support Care Cancer. 2024 Dec 18;33(1):40. doi: 10.1007/s00520-024-09087-w (PMC11655613; doi:10.1007/s00520-024-09087-w)
Supplement: Supplementary file 6 — Supplementary file6 (PDF 487 KB) [file 520_2024_9087_MOESM6_ESM.pdf]

S6. Distribution of Adverse Events

1 Death
2 Drug Interactions
3 Blood and lymphatic system disorders
4 Cardiac disorders
5 Congenital, familial and genetic disorders
6 Ear and labyrinth disorders
7 Endocrine disorders
8 Eye disorders
9 Gastrointestinal disorders
10 General disorders and administration site conditions
11 Hepatobiliary disorders
12 Immune system disorders
13 Infections and infestations
14 Injury, poisoning and procedural complications
15 Investigations
16 Metabolism and nutrition disorders
17 Musculoskeletal and connective tissue disorders
18 Neoplasms benign, malignant and unspecified (incl cysts and polyps)
19 Nervous system disorders
20 Psychiatric disorders
21 Renal and urinary disorders
22 Reproductive system and breast disorders
23 Respiratory, thoracic and mediastinal disorders
24 Skin and subcutaneous tissue disorders
25 Vascular disorders

Study ID	1	2	3	4	5	6	7	8	9	10	11	12	13	14	15	16	17	18	19	20	21	22	23	24	25
Abrahamov 1995(1)																				✓					
Ahmedzai 1983(2)	✓					✓		✓	✓	✓					✓				✓	✓					✓
Allen 2019(3)									✓	✓									✓						
Anderson 2019(4)									✓	✓						✓			✓	✓					
Aprikian 2023(5)				✓						✓			✓						✓						
Aviram 2020(6)	✓			✓		✓		✓	✓	✓						✓	✓		✓	✓					✓
Aviram 2022(7)				✓		✓		✓	✓	✓							✓		✓	✓			✓		✓
Bar-LevSchleider 2018(8)	✓								✓	✓						✓			✓	✓			✓		

Study ID	1	2	3	4	5	6	7	8	9	10	11	12	13	14	15	16	17	18	19	20	21	22	23	24	25
Bar-Sela 2013(9)																			✓						
Bar-Sela 2018(10)																									
Bar-Sela 2019(11)										✓									✓	✓					
Bar-Sela 2020(12)							✓			✓	✓	✓												✓	
Brisbois 2011(13)				✓					✓	✓			✓		✓	✓			✓	✓		✓	✓	✓	
Carr 2019(14)																			✓						
Cescon 2008(15)													✓												
Chan 1987(16)								✓								✓	✓		✓	✓					✓
Chang 1979(17)				✓															✓	✓					
Chang 1981(18)				✓																✓					
Chapman 2021(19)								✓	✓	✓						✓			✓						
Citron 1985(20)				✓				✓	✓	✓									✓	✓	✓				✓
Clarke 2022(21)									✓	✓									✓	✓			✓		
Colls 1980(22)																			✓	✓					
Cote 2016(23)									✓										✓	✓					

Study ID	1	2	3	4	5	6	7	8	9	10	11	12	13	14	15	16	17	18	19	20	21	22	23	24	25
Crawford 1986(24)									✓										✓	✓					
Cronin 1981(25)								✓	✓	✓							✓		✓	✓					✓
Cunningham 1988(26)								✓	✓										✓	✓					
Dall'Stella 2019(27)																								✓	
Dalzell 1986(28)								✓	✓							✓			✓	✓				✓	
D'Andre 2021(29)																			✓						
DAVIES 1974(30)				✓						✓									✓	✓					✓
Devine 1987(31)				✓					✓										✓	✓					✓
Diasio 1981(32)				✓					✓										✓	✓					✓
Dow 1984(33)				✓															✓	✓					✓
Duran 2010(34)				✓				✓	✓	✓									✓	✓			✓		
Einhorn 1981(35)																			✓	✓					✓
Ekert 1979(36)																✓			✓	✓					
Elliott 2016(37)																									
Fallon 2017(38)	✓								✓										✓	✓					

Study ID	1	2	3	4	5	6	7	8	9	10	11	12	13	14	15	16	17	18	19	20	21	22	23	24	25
Fehniger 2021(39)	✓								✓	✓									✓	✓					
Frytak 1979(40)								✓	✓										✓	✓					✓
Frytak 1984(41)																			✓	✓					
Gerhartz 1983(42)						✓													✓	✓					✓
Gilbert 1995(43)				✓																					
Good 2020(44)				✓					✓										✓	✓				✓	✓
Grimison 2020(45)			✓						✓	✓			✓		✓				✓	✓					✓
Guedon 2023(46)		✓	✓								✓								✓				✓		
Guy 2010(47)									✓										✓						
Guzman 2006(48)										✓										✓					
Hardy 2023(49)									✓	✓						✓			✓	✓			✓		
Hawley 2019(50)																									
Heim 1981(51)				✓				✓	✓	✓						✓			✓	✓					✓
Herman 1977(52)				✓					✓							✓			✓	✓					✓
Herman 1979(53)				✓				✓	✓										✓	✓					✓
Hsu 2020(54)										✓															

Study ID	1	2	3	4	5	6	7	8	9	10	11	12	13	14	15	16	17	18	19	20	21	22	23	24	25
Huniadi 2021(55)					✓																				
Hutcheon 1983(56)										✓									✓	✓					✓
Jafri 2023(57)																									
Jatoi 2002(58)	✓								✓	✓									✓	✓		✓			
Johnson 2010(59)	✓								✓	✓			✓		✓	✓		✓	✓	✓	✓				✓
Johnson 2013(60)	✓		✓						✓	✓	✓		✓	✓	✓	✓	✓	✓	✓	✓	✓		✓	✓	✓
Jones 1982(61)						✓		✓	✓	✓									✓	✓					✓
Joss 1982(62)				✓				✓	✓	✓									✓	✓					✓
Juarez 2021(63)		✓		✓					✓	✓			✓		✓	✓			✓	✓	✓			✓	
Kenyon 2018(64)																									
Khwaja 2016(65)	✓		✓					✓					✓		✓				✓				✓		
Kleinman 1983(66)																✓			✓	✓					
Klier 2021(67)																			✓	✓					
Kluin-Neleman 1979(68)				✓		✓		✓	✓	✓							✓		✓	✓				✓	
Kutiel 2018(69)																				✓					

Study ID	1	2	3	4	5	6	7	8	9	10	11	12	13	14	15	16	17	18	19	20	21	22	23	24	25
Lam 2023(70)									✓	✓															
Lane 1990(71)								✓	✓	✓									✓	✓			✓		
Lane 1991(72)				✓				✓	✓	✓									✓	✓			✓		✓
Laszlo 1981(73)				✓				✓	✓										✓	✓					✓
Levitt 1981(74)								✓																	
Li 2016(75)										✓									✓						✓
Lichtman 2018(76)	✓								✓	✓					✓	✓		✓	✓	✓					✓
Lucas 1980(77)									✓										✓	✓					✓
Lucraft 1982(78)									✓										✓	✓					
Lynch 2014(79)								✓	✓	✓						✓			✓	✓					
Macari 2020(80)									✓	✓									✓	✓					
Madden 2020(81)										✓									✓						
Maida 2008(82)																									
Maida 2008(83)									✓										✓	✓					
Maida 2017(84)	✓																								
Marchese 2022(85)																									

Study ID	1	2	3	4	5	6	7	8	9	10	11	12	13	14	15	16	17	18	19	20	21	22	23	24	25
McCabe 1988(86)																			✓	✓					
Meiri 2007(87)									✓	✓						✓			✓	✓					
Mekavuthikul 2020(88)				✓																					
Melen 2022(89)						✓			✓										✓	✓			✓		✓
Merkle 2018(90)																							✓		
Neidhart 1981(91)																			✓	✓					
Nelson 1994(92)									✓										✓	✓					
Niederle 1986(93)				✓					✓										✓	✓					✓
Nielsen 2022(94)								✓	✓	✓									✓	✓					
Nielsen 2022(95)									✓										✓						
Niiranen 1985(96)						✓			✓										✓	✓					✓
Niiranen 1987(97)						✓		✓	✓										✓	✓					✓
Noyes 1975(98)						✓		✓	✓							✓	✓		✓	✓				✓	✓
Noyes 1975(99)				✓		✓		✓								✓			✓	✓					✓
Ofir 2019(100)	✓								✓	✓										✓			✓		

Study ID	1	2	3	4	5	6	7	8	9	10	11	12	13	14	15	16	17	18	19	20	21	22	23	24	25
Orr 1980(101)																			✓	✓					
Parihar 2022(102)		✓																							
Philpot 2022(103)									✓							✓			✓						
Poghosyan 2021(104)																				✓					
Polito 2018(105)				✓				✓	✓	✓						✓	✓		✓	✓				✓	✓
Pomeroy 1986(106)									✓	✓									✓	✓					✓
Portenoy 2012(107)	✓		✓						✓	✓					✓	✓		✓	✓	✓					
Portman 2018(108)									✓																
Portman 2020(109)													✓												
Pralong 2018(110)																			✓						
Priestman 1984(111)									✓										✓						✓
Raghunathan 2022(112)									✓										✓	✓					✓
Reblin 2019(113)																									
Roffman 1986(114)																			✓	✓					
Russmann 2002(115)	✓									✓									✓						

Study ID	1	2	3	4	5	6	7	8	9	10	11	12	13	14	15	16	17	18	19	20	21	22	23	24	25
Saadeh 2018(116)		✓								✓									✓						
Sallan 1975(117)								✓											✓	✓					
Sallan 1980(118)																			✓	✓					
Sarid 2018(119)								✓		✓						✓			✓	✓					
Schloss 2021(120)								✓	✓	✓						✓	✓		✓	✓	✓		✓	✓	
Schmidt-Wolf 2021(121)	✓									✓						✓									
Senderovich 2022(122)									✓							✓			✓					✓	
Sheidler 1984(123)				✓				✓	✓	✓							✓		✓	✓				✓	✓
Singh 2013(124)									✓	✓					✓	✓			✓	✓					
Stambaugh 1984(125)																			✓	✓					✓
Staquet 1978(126)																			✓						
Steele 1980(127)									✓							✓			✓	✓			✓		✓
Strasser 2006(128)	✓		✓			✓			✓	✓						✓		✓	✓				✓		
Stuart-Harris 1983(129)									✓	✓									✓	✓				✓	✓
Sura 2022(130)																			✓	✓					

Study ID	1	2	3	4	5	6	7	8	9	10	11	12	13	14	15	16	17	18	19	20	21	22	23	24	25
Sutton 1986(131)	✓												✓												
Sweet 1981(132)				✓					✓										✓	✓					
Szyper-Kravitz 2001(133)													✓												
Taha 2019(134)							✓		✓	✓						✓	✓							✓	
Tavhare 2019(135)																			✓						
Tofthagen 2022(136)				✓					✓						✓				✓	✓			✓	✓	
Tschoe 2020(137)																			✓						
Turcott 2018(138)	✓									✓									✓						
Twelves 2021(139)	✓		✓					✓	✓	✓			✓		✓		✓	✓	✓	✓	✓		✓		
Tyson 1985(140)									✓	✓									✓	✓	✓				✓
Ungerleider 1982(141)				✓					✓	✓						✓			✓	✓					
Ungerleider 1985(142)										✓						✓			✓	✓					
vanHasselt 2012(143)																				✓					
Waissengrin 2015(144)										✓									✓	✓			✓		
Walsh 2005(145)									✓	✓									✓						

Study ID	1	2	3	4	5	6	7	8	9	10	11	12	13	14	15	16	17	18	19	20	21	22	23	24	25
Warren 2017(146)	✓								✓																
Webster 2020(147)				✓					✓							✓			✓	✓				✓	
Welsh 1983(148)																			✓	✓					
Zaki 2017(149)									✓										✓	✓					
Zhou 2021(150)																			✓	✓					
Zolotov 2021(151)									✓	✓									✓	✓			✓		
Zylla 2021(152)									✓										✓	✓					

References

1. Abrahamov A, Mechoulam R. AN EFFICIENT NEW CANNABINOID ANTIEMETIC IN PEDIATRIC ONCOLOGY. LIFE SCIENCES. 1995;56(23-24):2097-102.
2. Ahmedzai S, Carlyle DL, Calder IT, Moran F. Anti-emetic efficacy and toxicity of nabilone, a synthetic cannabinoid, in lung cancer chemotherapy. British journal of cancer. 1983;48(5):657-63.
3. Allen D. Dronabinol Therapy: Central Nervous System Adverse Events in Adults With Primary Brain Tumors. Clinical journal of oncology nursing. 2019;23(1):23-6.
4. Anderson SP, Zylla DM, McGriff DM, Arneson TJ. Impact of Medical Cannabis on Patient-Reported Symptoms for Patients With Cancer Enrolled in Minnesota's Medical Cannabis Program. Journal of oncology practice. 2019;15(4):e338-e45.
5. Aprikian S, Kasvis P, Vigano M, Hachem Y, Canac-Marquis M, Vigano A. Medical cannabis is effective for cancer-related pain: Quebec Cannabis Registry results. BMJ supportive & palliative care. 2023(101565123).
6. Aviram J, Lewitus GM, Vysotski Y, Uribayev A, Procaccia S, Cohen I, et al. Short-Term Medical Cannabis Treatment Regimens Produced Beneficial Effects among Palliative Cancer Patients. Pharmaceuticals (Basel, Switzerland). 2020;13(12).
7. Aviram J, Lewitus GM, Vysotski Y, Amna MA, Ouryvaev A, Procaccia S, et al. The Effectiveness and Safety of Medical Cannabis for Treating Cancer Related Symptoms in Oncology Patients. Frontiers in pain research (Lausanne, Switzerland). 2022;3(9918227269806676):861037.

8. Bar-Lev Schleider L, Mechoulam R, Lederman V, Hilou M, Lencovsky O, Betzalel O, et al. Prospective analysis of safety and efficacy of medical cannabis in large unselected population of patients with cancer. European journal of internal medicine. 2018;49(9003220):37-43.
9. Bar-Sela G, Vorobeichik M, Drawsheh S, Omer A, Goldberg V, Muller E. The medical necessity for medicinal cannabis: prospective, observational study evaluating the treatment in cancer patients on supportive or palliative care. Evidence-based complementary and alternative medicine : eCAM. 2013;2013(101215021):510392.
10. Bar-Sela G, Tauber D, Mitnik I, Sheinman-Yuffe H, Bishara-Frolova T, Aharon-Peretz J. Cannabis-related cognitive impairment: A prospective evaluation of possible influences on patients with cancer during chemotherapy treatment as a pilot study. Anti-Cancer Drugs. 2018;30(1):91-7.
11. Bar-Sela G, Zalman D, Semenysty V, Ballan E. The Effects of Dosage-Controlled Cannabis Capsules on Cancer-Related Cachexia and Anorexia Syndrome in Advanced Cancer Patients: Pilot Study. Integrative cancer therapies. 2019;18(101128834):1534735419881498.
12. Bar-Sela G, Cohen I, Campisi-Pinto S, Lewitus GM, Oz-Ari L, Jehassi A, et al. Cannabis Consumption Used by Cancer Patients during Immunotherapy Correlates with Poor Clinical Outcome. Cancers. 2020;12(9).
13. Brisbois TD, de Kock IH, Watanabe SM, Mirhosseini M, Lamoureux DC, Chasen M, et al. Delta-9-tetrahydrocannabinol may palliate altered chemosensory perception in cancer patients: results of a randomized, double-blind, placebo-controlled pilot trial. Annals of oncology : official journal of the European Society for Medical Oncology. 2011;22(9):2086-93.
14. Carr C, Vertelney H, Fronk J, Trieu S. Dronabinol for the Treatment of Paraneoplastic Night Sweats in Cancer Patients: A Report of Five Cases. Journal of Palliative Medicine. 2019;22(10):1221-3.
15. Cescon DW, Page AV, Richardson S, Moore MJ, Boerner S, Gold WL. Invasive pulmonary aspergillosis associated with marijuana use in a man with colorectal cancer. Journal of clinical oncology : official journal of the American Society of Clinical Oncology. 2008;26(13):2214-5.
16. Chan HS, Correia JA, MacLeod SM. Nabilone versus prochlorperazine for control of cancer chemotherapy-induced emesis in children: a double-blind, crossover trial. Pediatrics. 1987;79(6):946-52.
17. Chang AE, Shiling DJ, Stillman RC. Delta-9-tetrahydrocannabinol as an antiemetic in cancer patients receiving high-dose methotrexate. A prospective, randomized evaluation. Annals of Internal Medicine. 1979;91(6):819-24.
18. Chang AE, Shiling DJ, Stillman RC, Goldberg NH, Seipp CA, Barofsky I, et al. A prospective evaluation of delta-9-tetrahydrocannabinol as an antiemetic in patients receiving adriamycin and cytoxan chemotherapy. Cancer. 1981;47(7):1746-51.
19. Chapman S, Protudjer J, Bourne C, Kelly LE, Oberoi S, Vanan MI. Medical cannabis in pediatric oncology: a survey of patients and caregivers. Supportive care in cancer : official journal of the Multinational Association of Supportive Care in Cancer. 2021;29(11):6589-94.
20. Citron ML, Herman TS, Vreeland F, Krasnow SH, Fossieck BE, Jr., Harwood S, et al. Antiemetic efficacy of levonantradol compared to delta-9-tetrahydrocannabinol for chemotherapy-induced nausea and vomiting. Cancer treatment reports. 1985;69(1):109-12.
21. Clarke S, Butcher BE, McLachlan AJ, Henson JD, Rutolo D, Hall S, et al. Pilot clinical and pharmacokinetic study of Δ9-Tetrahydrocannabinol (THC)/Cannabidiol (CBD) nanoparticle oro-buccal spray in patients with advanced cancer experiencing uncontrolled pain. PLoS ONE. 2022;17(10 October).
22. Colls BM, Ferry DG, Gray AJ, Harvey VJ, McQueen EG. The antiemetic activity of tetrahydrocannabinol versus metoclopramide and thiethylperazine in patients undergoing cancer chemotherapy. The New Zealand medical journal. 1980;91(662):449-51.

23. Cote M, Trudel M, Wang C, Fortin A. Improving Quality of Life With Nabilone During Radiotherapy Treatments for Head and Neck Cancers: A Randomized Double-Blind Placebo-Controlled Trial. The Annals of otology, rhinology, and laryngology. 2016;125(4):317-24.
24. Crawford SM, Buckman R. Nabilone and metoclopramide in the treatment of nausea and vomiting due to cisplatinum: A double blind study. Medical Oncology and Tumor Pharmacotherapy. 1986;3(1):39-42.
25. Cronin CM, Sallan SE, Gelber R, Lucas VS, Laszlo J. Antiemetic effect of intramuscular levonantradol in patients receiving anticancer chemotherapy. Journal of clinical pharmacology. 1981;21(S1):43S-50S.
26. Cunningham D, Bradley CJ, Forrest GJ, Hutcheon AW, Adams L, Sneddon M, et al. A randomized trial of oral nabilone and prochlorperazine compared to intravenous metoclopramide and dexamethasone in the treatment of nausea and vomiting induced by chemotherapy regimens containing cisplatin or cisplatin analogues. European journal of cancer & clinical oncology. 1988;24(4):685-9.
27. Dall'Stella PB, Docema MFL, Maldaun MVC, Feher O, Lancellotti CLP. Case report: Clinical outcome and image response of two patients with secondary high-grade glioma treated with chemoradiation, PCV, and cannabidiol. Frontiers in Oncology. 2019;9(JAN):643.
28. Dalzell AM, Bartlett H, Lilleyman JS. Nabilone: an alternative antiemetic for cancer chemotherapy. Archives of disease in childhood. 1986;61(5):502-5.
29. D'Andre S, McAllister S, Nagi J, Giridhar KV, Ruiz-Macias E, Loprinzi C. Topical Cannabinoids for Treating Chemotherapy-Induced Neuropathy: A Case Series. Integrative cancer therapies. 2021;20(101128834):15347354211061739.
30. Davies BH, Weatherstone RM, Graham JDP, Griffiths RD. A pilot study of orally administered Δ1-trans-tetrahydrocannabinol in the management of patients undergoing radiotherapy for carcinoma of the bronchus. Br J Clin Pharmacol. 1974;1(4):301-6.
31. Devine ML, Dow GJ, Greenberg BR. Adverse reactions to delta-9-tetrahydrocannabinol given as an antiemetic in a multicenter study. Clinical Pharmacy. 1987;6(4):319-22.
32. Diasio RB, Ettinger DS, Satterwhite BE. Oral Levonantradol in the Treatment of Chemotherapy-Induced Emesis: Preliminary Observations. J Clin Pharmacol. 1981;21(1 S):81S-5S.
33. Dow GJ, Meyers FH, Stanton W, Devine ML. Serious reactions to oral delta-9-tetrahydrocannabinol in cancer chemotherapy patients. Clinical pharmacy. 1984;3(1):14.
34. Duran M, Perez E, Abanades S, Vidal X, Saura C, Majem M, et al. Preliminary efficacy and safety of an oromucosal standardized cannabis extract in chemotherapy-induced nausea and vomiting. British journal of clinical pharmacology. 2010;70(5):656-63.
35. Einhorn LH, Nagy C, Furnas B, Williams SD. Nabilone: an effective antiemetic in patients receiving cancer chemotherapy. Journal of clinical pharmacology. 1981;21(S1):64S-9S.
36. Ekert H, Waters KD, Jurk IH, Mobilia J, Loughnan P. Amelioration of cancer chemotherapy-induced nausea and vomiting by delta-9-tetrahydrocannabinol. The Medical journal of Australia. 1979;2(12):657-9.
37. Elliott DA, Nabavizadeh N, Romer JL, Chen Y, Holland JM. Medical marijuana use in head and neck squamous cell carcinoma patients treated with radiotherapy. Supportive care in cancer : official journal of the Multinational Association of Supportive Care in Cancer. 2016;24(8):3517-24.

38. Fallon MT, Albert Lux E, McQuade R, Rossetti S, Sanchez R, Sun W, et al. Sativex oromucosal spray as adjunctive therapy in advanced cancer patients with chronic pain unalleviated by optimized opioid therapy: two double-blind, randomized, placebo-controlled phase 3 studies. British journal of pain. 2017;11(3):119-33.
39. Fehniger J, Brodsky AL, Kim A, Pothuri B. Medical marijuana utilization in gynecologic cancer patients. Gynecologic oncology reports. 2021;37(101652231):100820.
40. Frytak S, Moertel CG, O'Fallon JR, Rubin J, Creagan ET, O'Connell MJ, et al. Delta-9-tetrahydrocannabinol as an antiemetic for patients receving cancer chemotherapy. A comparison with prochlorperazine and a placebo. Annals of Internal Medicine. 1979;91(6):825-30.
41. Frytak S, Moertel CG, Rubin J. Metabolic studies of delta-9-tetrahydrocannabinol in cancer patients. Cancer Treatment Reports. 1984;68(12):1427-31.
42. Gerhartz HH, Binsack T, Hiller E. Levonantradol for the treatment of chemotherapy-induced nausea and vomiting. Klinische Wochenschrift. 1983;61(14):719-21.
43. Gilbert CJ, Ohly KV, Rosner G, Peters WP. Randomized, double-blind comparison of a prochlorperazine-based versus a metoclopramide-based antiemetic regimen in patients undergoing autologous bone marrow transplantation. Cancer. 1995;76(11):2330-7.
44. Good PD, Greer RM, Huggett GE, Hardy JR. An Open-Label Pilot Study Testing the Feasibility of Assessing Total Symptom Burden in Trials of Cannabinoid Medications in Palliative Care. Journal of palliative medicine. 2020;23(5):650-5.
45. Grimison P, Mersiades A, Kirby A, Lintzeris N, Morton R, Haber P, et al. Oral THC:CBD cannabis extract for refractory chemotherapy-induced nausea and vomiting: a randomised, placebo-controlled, phase II crossover trial. Annals of oncology : official journal of the European Society for Medical Oncology. 2020;31(11):1553-60.
46. Guedon M, Le Bozec A, Brugel M, Clarenne J, Carlier C, Perrier M, et al. Cannabidiol-drug interaction in cancer patients: A retrospective study in a real-life setting. British journal of clinical pharmacology. 2023(au9, 7503323).
47. Guy G, Gover J, Rogerson M, Atwell B, Dineen J. Positive data in sativex phase IIb trial: Support advancing into phase III development in cancer pain. Revista de la Sociedad Espanola del Dolor. 2010;17(4):219-21.
48. Guzman M, Duarte MJ, Blazquez C, Ravina J, Rosa MC, Galve-Roperh I, et al. A pilot clinical study of Delta9-tetrahydrocannabinol in patients with recurrent glioblastoma multiforme. British journal of cancer. 2006;95(2):197-203.
49. Hardy J, Greer R, Huggett G, Kearney A, Gurgenci T, Good P. Phase IIb Randomized, Placebo-Controlled, Dose-Escalating, Double-Blind Study of Cannabidiol Oil for the Relief of Symptoms in Advanced Cancer (MedCan1-CBD). Journal of clinical oncology : official journal of the American Society of Clinical Oncology. 2023;41(7):1444-52.
50. Hawley P, Gobbo M. Cannabis use in cancer: a survey of the current state at BC Cancer before recreational legalization in Canada. Current oncology (Toronto, Ont). 2019;26(4):e425-e32.
51. Heim ME, Romer W, Queisser W. Clinical experience with levonantradol hydrochloride in the prevention of cancer chemotherapy-induced nausea and vomiting. Journal of clinical pharmacology. 1981;21(S1):86S-9S.
52. Herman TS, Jones SE, Dean J, Leigh S, Dorr R, Moon TE, et al. Nabilone: a potent antiemetic cannabinol with minimal euphoria. Biomedicine / [publiee pour l'AAICIG]. 1977;27(9-10):331-4.

53. Herman TS, Einhorn LH, Jones SE, Nagy C, Chester AB, Dean JC, et al. Superiority of nabilone over prochlorperazine as an antiemetic in patients receiving cancer chemotherapy. The New England journal of medicine. 1979;300(23):1295-7.
54. Hsu K, Whitham E, Kichenadasse G. Potential role of cannabidiol for seizure control in a patient with recurrent glioma. Journal of clinical neuroscience : official journal of the Neurosurgical Society of Australasia. 2020;71(dpi, 9433352):275-6.
55. Huniadi A, Sorian A, Iuhas C, Bodog A, Sandor MI. The effect of cannabis in the treatment of hodgkin's lymphoma in a pregnant patient - extensive case report and literature review. Journal of BUON. 2021;26(1):11-6.
56. Hutcheon AW, Palmer JB, Soukop M, Cunningham D, McArdle C, Welsh J, et al. A randomised multicentre single blind comparison of a cannabinoid anti-emetic (levonantradol) with chlorpromazine in patients receiving their first cytotoxic chemotherapy. European journal of cancer & clinical oncology. 1983;19(8):1087-90.
57. Jafri S, Hansen E, Fuenmayor R, Case AA. Medical Cannabis for Insomnia in a Patient With Advanced Breast Cancer. Journal of Pain and Symptom Management. 2023;65(5):e497-e502.
58. Jatoi A, Windschitl HE, Loprinzi CL, Sloan JA, Dakhil SR, Mailliard JA, et al. Dronabinol versus megestrol acetate versus combination therapy for cancer-associated anorexia: a North Central Cancer Treatment Group study. Journal of clinical oncology : official journal of the American Society of Clinical Oncology. 2002;20(2):567-73.
59. Johnson JR, Burnell-Nugent M, Lossignol D, Ganae-Motan ED, Potts R, Fallon MT. Multicenter, double-blind, randomized, placebo-controlled, parallel-group study of the efficacy, safety, and tolerability of THC:CBD extract and THC extract in patients with intractable cancer-related pain. Journal of pain and symptom management. 2010;39(2):167-79.
60. Johnson JR, Lossignol D, Burnell-Nugent M, Fallon MT. An open-label extension study to investigate the long-term safety and tolerability of THC/CBD oromucosal spray and oromucosal THC spray in patients with terminal cancer-related pain refractory to strong opioid analgesics. Journal of pain and symptom management. 2013;46(2):207-18.
61. Jones SE, Durant JR, Greco FA, Robertone A. A multi-institutional phase III study of nabilone vs. placebo in chemotherapy-induced nausea and vomiting. Cancer Treatment Reviews. 1982;9(Suppl. B):45-8.
62. Joss RA, Galeazzi RL, Bischoff A, Do DD, Goldhirsch A, Brunner KW. Levonantradol, a new antiemetic with a high rate of side-effects for the prevention of nausea and vomiting in patients receiving cancer chemotherapy. Cancer chemotherapy and pharmacology. 1982;9(1):61-4.
63. Juarez TM, Piccioni D, Rose L, Nguyen A, Brown B, Kesari S. Phase I dose-escalation, safety, and CNS pharmacokinetic study of dexanabinol in patients with brain cancer. Neuro-oncology advances. 2021;3(1):vdab006.
64. Kenyon J, Liu W, Dalgleish A. Report of Objective Clinical Responses of Cancer Patients to Pharmaceutical-grade Synthetic Cannabidiol. Anticancer research. 2018;38(10):5831-5.
65. Khwaja S, Yacoub A, Cheema A, Rihana N, Russo R, Velez AP, et al. Marijuana Smoking in Patients With Leukemia. Cancer control : journal of the Moffitt Cancer Center. 2016;23(3):278-83.
66. Kleinman S, Weitzman SA, Cassem N, Andrews E. Double blind trial of delta-9-tetrahydrocannabinol (THC) versus placebo as an adjunct to prochlorperazine for chemotherapy-induced vomiting. Current Therapeutic Research - Clinical and Experimental. 1983;33(6 I):1014-7.

67. Klier CM, Amminger GP, Kothgassner OD, Laczkovics C, Felnhofer A. Letter to the Editor: Cannabidiol Treatment - Is There an Effect on Cognitive Functioning, Quality of Life, and Behavior? A Case Report. Journal of Child and Adolescent Psychopharmacology. 2021;31(6):447-9.
68. Kluin-Neleman JC, Neleman FA, Meuwissen OJ, Maes RA. delta 9-Tetrahydrocannabinol (THC) as an antiemetic in patients treated with cancerchemotherapy; a double-blind cross-over trial against placebo. Veterinary and human toxicology. 1979;21(5):338-40.
69. Kutiel TS, Vornicova O, Bar-Sela G. Cannabis for Vismodegib-Related Muscle Cramps in a Patient With Advanced Basal Cell Carcinoma. Journal of pain and symptom management. 2018;55(5):e1-e2.
70. Lam CS, Zhou K, Loong HH-F, Chung VC-H, Ngan C-K, Cheung YT. The Use of Traditional, Complementary, and Integrative Medicine in Cancer: Data-Mining Study of 1 Million Web-Based Posts From Health Forums and Social Media Platforms. Journal of medical Internet research. 2023;25(100959882):e45408.
71. Lane M, Smith FE, Sullivan RA, Plasse TF. Dronabinol and prochlorperazine alone and in combination as antiemetic agents for cancer chemotherapy. American journal of clinical oncology. 1990;13(6):480-4.
72. Lane M, Vogel CL, Ferguson J, Krasnow S, Saiers JL, Hamm J, et al. Dronabinol and prochlorperazine in combination for treatment of cancer chemotherapy-induced nausea and vomiting. Journal of pain and symptom management. 1991;6(6):352-9.
73. Laszlo J, Lucas VS, Jr., Hanson DC, Cronin CM, Sallan SE. Levonantradol for chemotherapy-induced emesis: phase I-II oral administration. Journal of clinical pharmacology. 1981;21(S1):51S-6S.
74. Levitt M, Wilson A, Bowman D, Kemel S, Krepart G, Marks V, et al. Physiologic observations in a controlled clinical trial of the antiemetic effectiveness of 5, 10, and 15 mg of delta 9-tetrahydrocannabinol in cancer chemotherapy. Ophthalmologic implications. Journal of clinical pharmacology. 1981;21(S1):103S-9S.
75. Li AM, Rassekh SR. Hypotension associated with ingestion of cannabinoids in two children with cancer. CMAJ. 2016;188(8):596-7.
76. Lichtman AH, Lux EA, McQuade R, Rossetti S, Sanchez R, Sun W, et al. Results of a Double-Blind, Randomized, Placebo-Controlled Study of Nabiximols Oromucosal Spray as an Adjunctive Therapy in Advanced Cancer Patients with Chronic Uncontrolled Pain. Journal of pain and symptom management. 2018;55(2):179-88.e1.
77. Lucas VS, Jr., Laszlo J. delta 9-Tetrahydrocannabinol for refractory vomiting induced by cancer chemotherapy. JAMA. 1980;243(12):1241-3.
78. Lucraft HH, Palmer MK. Randomised clinical trial of levonantradol and chlorpromazine in the prevention of radiotherapy-induced vomiting. Clinical radiology. 1982;33(6):621-2.
79. Lynch ME, Cesar-Rittenberg P, Hohmann AG. A double-blind, placebo-controlled, crossover pilot trial with extension using an oral mucosal cannabinoid extract for treatment of chemotherapy-induced neuropathic pain. Journal of pain and symptom management. 2014;47(1):166-73.
80. Macari DM, Gbadamosi B, Jaiyesimi I, Gaikazian S. Medical Cannabis in Cancer Patients: A Survey of a Community Hematology Oncology Population. American journal of clinical oncology. 2020;43(9):636-9.
81. Madden K, Tanco K, Bruera E. Clinically Significant Drug-Drug Interaction Between Methadone and Cannabidiol. Pediatrics. 2020;145(6).
82. Maida V. Nabilone for the treatment of paraneoplastic night sweats: A report of four cases. Journal of Palliative Medicine. 2008;11(6):929-34.
83. Maida V, Ennis M, Irani S, Corbo M, Dolzhykov M. Adjunctive nabilone in cancer pain and symptom management: A prospective observational study using propensity scoring. Journal of Supportive Oncology. 2008;6(3):119-24.

84. Maida V. Medical Cannabis in the Palliation of Malignant Wounds-A Case Report. Journal of Pain and Symptom Management. 2017;53(1):e4-e6.
85. Marchese M, Zhu C, Charbonneau LF, Peragine C, De Angelis C. Description and Disposition of Patients With Cancer Accessing a Novel, Pharmacist-Led Cannabis Consultation Service. JCO Oncology Practice. 2022;313((Marchese, Charbonneau, Peragine, De Angelis) Department of Pharmacy, Sunnybrook Odette Cancer Centre, Toronto, Canada(Marchese, Zhu, De Angelis) Leslie Dan Faculty of Pharmacy, University of Toronto, Toronto, Canada(De Angelis) Sunnybrook Research Instit):00748.
86. McCabe M, Smith FP, Macdonald JS, Woolley PV, Goldberg D, Schein PS. Efficacy of tetrahydrocannabinol in patients refractory to standard antiemetic therapy. Investigational new drugs. 1988;6(3):243-6.
87. Meiri E, Jhangiani H, Vredenburgh JJ, Barbato LM, Carter FJ, Yang H-M, et al. Efficacy of dronabinol alone and in combination with ondansetron versus ondansetron alone for delayed chemotherapy-induced nausea and vomiting. Current medical research and opinion. 2007;23(3):533-43.
88. Mekavuthikul P, Phuditshinnapatra J, Chomchai S. Cannabis oil could provoke secondary myocardial infarction in a high-risk patient after dropping into both ear and sublingual administration: A case report. Clinical Toxicology. 2020;58(4):354.
89. Melen CM, Merrien M, Wasik AM, Panagiotidis G, Beck O, Sonnevi K, et al. Clinical effects of a single dose of cannabinoids to patients with chronic lymphocytic leukemia. Leukemia & lymphoma. 2022;63(6):1387-97.
90. Merkle S, Tavernier SS. Cannabis Use and Bleomycin: An Overview and Case Study of Pulmonary Toxicity. Clinical journal of oncology nursing. 2018;22(4):438-43.
91. Neidhart JA, Gagen MM, Wilson HE, Young DC. Comparative trial of the antiemetic effects of THC and haloperidol. Journal of clinical pharmacology. 1981;21(S1):38S-42S.
92. Nelson K, Walsh D, Deeter P, Sheehan F. A phase II study of delta-9-tetrahydrocannabinol for appetite stimulation in cancer-associated anorexia. Journal of palliative care. 1994;10(1):14-8.
93. Niederle N, Schutte J, Schmidt CG. Crossover comparison of the antiemetic efficacy of nabilone and alizapride in patients with nonseminomatous testicular cancer receiving cisplatin therapy. Klinische Wochenschrift. 1986;64(8):362-5.
94. Nielsen SW, Ruhlmann CH, Eckhoff L, Bronnum D, Herrstedt J, Dalton SO. Cannabis use among Danish patients with cancer: a cross-sectional survey of sociodemographic traits, quality of life, and patient experiences. Supportive care in cancer : official journal of the Multinational Association of Supportive Care in Cancer. 2022;30(2):1181-90.
95. Nielsen SW, Hasselsteen SD, Dominiak HSH, Labudovic D, Reiter L, Dalton SO, et al. Oral cannabidiol for prevention of acute and transient chemotherapy-induced peripheral neuropathy. Supportive care in cancer : official journal of the Multinational Association of Supportive Care in Cancer. 2022;30(11):9441-51.
96. Niiranen A, Mattson K. A cross-over comparison of nabilone and prochlorperazine for emesis induced by cancer chemotherapy. American journal of clinical oncology. 1985;8(4):336-40.
97. Niiranen A, Mattson K. Antiemetic efficacy of nabilone and dexamethasone: a randomized study of patients with lung cancer receiving chemotherapy. American journal of clinical oncology. 1987;10(4):325-9.

98. Noyes R, Jr., Brunk SF, Avery DA, Canter AC. The analgesic properties of delta-9-tetrahydrocannabinol and codeine. Clinical pharmacology and therapeutics. 1975;18(1):84-9.
99. Noyes R, Jr., Brunk SF, Baram DA, Canter A. Analgesic effect of delta-9-tetrahydrocannabinol. Journal of clinical pharmacology. 1975;15(2-3):139-43.
100. Ofir R, Bar-Sela G, Weyl Ben-Arush M, Postovsky S. Medical marijuana use for pediatric oncology patients: single institution experience. Pediatric hematology and oncology. 2019;36(5):255-66.
101. Orr LE, McKernan JF, Bloome B. Antiemetic effect of tetrahydrocannabinol. Compared with placebo and prochlorperazine in chemotherapy-associated nausea and emesis. Archives of internal medicine. 1980;140(11):1431-3.
102. Parihar V, Rogers A, Blain AM, Zacharias SRK, Patterson LL, Siyam MA-M. Reduction in Tamoxifen Metabolites Endoxifen and N-desmethyltamoxifen With Chronic Administration of Low Dose Cannabidiol: A CYP3A4 and CYP2D6 Drug Interaction. Journal of pharmacy practice. 2022;35(2):322-6.
103. Philpot LM, Ramar P, Jatoi A, Rosedahl J, Canning R, Ebbert JO. Cannabis in Cancer Survivors Who Report High Impact Chronic Pain: Findings from a 1500+ Patient Survey. The American journal of hospice & palliative care. 2022(9008229, atd):10499091221143098.
104. Poghosyan H, Noonan EJ, Badri P, Braun I, Young GJ. Association between daily and non-daily cannabis use and depression among United States adult cancer survivors. Nursing outlook. 2021;69(4):672-85.
105. Polito S, MacDonald T, Romanick M, Jupp J, Wiernikowski J, Vennettilli A, et al. Safety and efficacy of nabilone for acute chemotherapy-induced vomiting prophylaxis in pediatric patients: A multicenter, retrospective review. Pediatric blood & cancer. 2018;65(12):e27374.
106. Pomeroy M, Fennelly JJ, Towers M. Prospective randomized double-blind trial of nabilone versus domperidone in the treatment of cytotoxic-induced emesis. Cancer chemotherapy and pharmacology. 1986;17(3):285-8.
107. Portenoy RK, Ganae-Motan ED, Allende S, Yanagihara R, Shaiova L, Weinstein S, et al. Nabiximols for opioid-treated cancer patients with poorly-controlled chronic pain: a randomized, placebo-controlled, graded-dose trial. The journal of pain. 2012;13(5):438-49.
108. Portman D, Donovan KA. Cannabinoid Hyperemesis Syndrome: A Case Report of a Confounding Entity in Oncology Care. Journal of oncology practice. 2018;14(5):333-4.
109. Portman D, Donovan KA. Case of Pneumonitis Associated With Cannabis Vaping and Cancer Immunotherapy. JCO oncology practice. 2020;16(5):245-6.
110. Pralong E, Maduri R, Daniel RT, Messerer M. Cannabinoid-induced alteration of motor-evoked potentials (MEPs) prior to intradural spinal tumor removal: a nasty surprise. Child's nervous system : ChNS : official journal of the International Society for Pediatric Neurosurgery. 2018;34(7):1287-9.
111. Priestman TJ, Priestman SG. An initial evaluation of Nabilone in the control of radiotherapy-induced nausea and vomiting. Clinical radiology. 1984;35(4):265-6.
112. Raghunathan NJ, Brens J, Vemuri S, Li QS, Mao JJ, Korenstein D. In the weeds: a retrospective study of patient interest in and experience with cannabis at a cancer center. Supportive care in cancer : official journal of the Multinational Association of Supportive Care in Cancer. 2022;30(9):7491-7.

113. Reblin M, Sahebjam S, Peeri NC, Martinez YC, Thompson Z, Egan KM. Medical Cannabis Use in Glioma Patients Treated at a Comprehensive Cancer Center in Florida. Journal of Palliative Medicine. 2019;22(10):1202-7.
114. Roffman RA. Stress inoculation training in the control of THC toxicities. The International journal of the addictions. 1986;21(8):883-96.
115. Russmann S, Winkler A, Lovblad KO, Stanga Z, Bassetti C. Lethal ischemic stroke after cisplatin-based chemotherapy for testicular carcinoma and cannabis inhalation. European neurology. 2002;48(3):178-80.
116. Saadeh CE, Rustem DR. Medical Marijuana Use in a Community Cancer Center. Journal of oncology practice. 2018;14(9):e566-e78.
117. Sallan SE, Zinberg NE, Frei E, 3rd. Antiemetic effect of delta-9-tetrahydrocannabinol in patients receiving cancer chemotherapy. The New England journal of medicine. 1975;293(16):795-7.
118. Sallan SE, Cronin C, Zelen M, Zinberg NE. Antiemetics in patients receiving chemotherapy for cancer: a randomized comparison of delta-9-tetrahydrocannabinol and prochlorperazine. The New England journal of medicine. 1980;302(3):135-8.
119. Sarid N, Zada M, Lev-Ran S, Yashphe E, Givon I, Barzilai M, et al. Medical Cannabis Use by Hodgkin Lymphoma Patients: Experience of a Single Center. Acta haematologica. 2018;140(4):194-202.
120. Schloss J, Lacey J, Sinclair J, Steel A, Sughrue M, Sibbritt D, et al. A Phase 2 Randomised Clinical Trial Assessing the Tolerability of Two Different Ratios of Medicinal Cannabis in Patients With High Grade Gliomas. Frontiers in oncology. 2021;11(101568867):649555.
121. Schmidt-Wolf G, Cremer-Schaeffer P. Interim results of the survey accompanying the prescription of cannabis-based medicines in Germany regarding dronabinol. Deutsches Arzteblatt International. 2021;118(10):177-8.
122. Senderovich H, Waicus S. A Case Report on Cannabinoid Hyperemesis Syndrome in Palliative Care: How Good Intentions Can Go Wrong. Oncology research and treatment. 2022;45(7-8):438-43.
123. Sheidler VR, Ettinger DS, Diasio RB, Enterline JP, Brown MD. Double-blind multiple-dose crossover study of the antiemetic effect of intramuscular levonantradol compared to prochlorperazine. Journal of clinical pharmacology. 1984;24(4):155-9.
124. Singh Y, Bali C. Cannabis extract treatment for terminal acute lymphoblastic leukemia with a Philadelphia chromosome mutation. Case Reports in Oncology. 2013;6(3):585-92.
125. Stambaugh JE, Jr., McAdams J, Vreeland F. Dose ranging evaluation of the antiemetic efficacy and toxicity of intramuscular levonantradol in cancer subjects with chemotherapy-induced emesis. Journal of clinical pharmacology. 1984;24(11-12):480-5.
126. Staquet M, Gantt C, Machin D. Effect of a nitrogen analog of tetrahydrocannabinol on cancer pain. Clinical pharmacology and therapeutics. 1978;23(4):397-401.
127. Steele N, Gralla RJ, Braun DW, Jr., Young CW. Double-blind comparison of the antiemetic effects of nabilone and prochlorperazine on chemotherapy-induced emesis. Cancer treatment reports. 1980;64(2-3):219-24.
128. Strasser F, Luftner D, Possinger K, Ernst G, Ruhstaller T, Meissner W, et al. Comparison of orally administered cannabis extract and delta-9-tetrahydrocannabinol in treating patients with cancer-related anorexia-cachexia syndrome: a multicenter, phase III, randomized, double-blind, placebo-controlled clinical trial from the Cannabi. Journal of clinical oncology : official journal of the American Society of Clinical Oncology. 2006;24(21):3394-400.

129. Stuart-Harris RC, Mooney CA, Smith IE. Levonantradol: a synthetic cannabinoid in the treatment of severe chemotherapy-induced nausea and vomiting resistant to conventional anti-emetic therapy. Clinical oncology. 1983;9(2):143-6.
130. Sura KT, Kohman L, Huang D, Pasniciuc SV. Experience With Medical Marijuana for Cancer Patients in the Palliative Setting. Cureus. 2022;14(6):e26406.
131. Sutton S, Lum BL, Torti FM. Possible risk of invasive pulmonary aspergillosis with marijuana use during chemotherapy for small cell lung cancer. Drug intelligence & clinical pharmacy. 1986;20(4):289-91.
132. Sweet DL, Miller NJ, Weddington W, Senay E, Sushelsky L. delta 9-Tetrahydrocannabinol as an antiemetic for patients receiving cancer chemotherapy. A pilot study. Journal of clinical pharmacology. 1981;21(S1):70S-5S.
133. Szyper-Kravitz M, Lang R, Manor Y, Lahav M. Early invasive pulmonary aspergillosis in a leukemia patient linked to aspergillus contaminated marijuana smoking. Leukemia & lymphoma. 2001;42(6):1433-7.
134. Taha T, Meiri D, Talhamy S, Wollner M, Peer A, Bar-Sela G. Cannabis Impacts Tumor Response Rate to Nivolumab in Patients with Advanced Malignancies. The oncologist. 2019;24(4):549-54.
135. Tavhare SD, Acharya R, Reddy RG, Dhiman KS. Management of chronic pain with Jalaprakshalana (water-wash) Shodhita (processed) Bhanga (Cannabis sativa L.) in cancer patients with deprived quality of life: An open-label single arm clinical trial. Ayu. 2019;40(1):34-43.
136. Tofthagen C, Perlman A, Advani P, Ernst B, Kaur J, Tan W, et al. Medical Marijuana Use for Cancer-Related Symptoms among Floridians: A Descriptive Study. Journal of palliative medicine. 2022;25(10):1563-70.
137. Tschoe C, Johnson L, Giugliano A, Sarwal A. Serotonin syndrome with exposure from tetrahydrocannabinol: a case report to highlight the side effects of increasing use of cbd products. Neurology. 2020;94(15 Supplement).
138. Turcott JG, del Rocio Guillen Nunez M, Flores-Estrada D, Onate-Ocana LF, Zatarain-Barron ZL, Barron F, et al. The effect of nabilone on appetite, nutritional status, and quality of life in lung cancer patients: a randomized, double-blind clinical trial. Supportive Care in Cancer. 2018;26(9):3029-38.
139. Twelves C, Sabel M, Checketts D, Miller S, Tayo B, Jove M, et al. A phase 1b randomised, placebo-controlled trial of nabiximols cannabinoid oromucosal spray with temozolomide in patients with recurrent glioblastoma. British journal of cancer. 2021;124(8):1379-87.
140. Tyson LB, Gralla RJ, Clark RA, Kris MG, Bordin LA, Bosl GJ. Phase 1 trial of levonantradol in chemotherapy-induced emesis. American journal of clinical oncology. 1985;8(6):528-32.
141. Ungerleider JT, Andrysiak T, Fairbanks L, Goodnight J, Sarna G, Jamison K. Cannabis and cancer chemotherapy: a comparison of oral delta-9-THC and prochlorperazine. Cancer. 1982;50(4):636-45.
142. Ungerleider JT, Sarna G, Fairbanks LA, Goodnight J, Andrysiak T, Jamison K. THC or Compazine for the cancer chemotherapy patient--the UCLA study. Part II: Patient drug preference. American journal of clinical oncology. 1985;8(2):142-7.
143. van Hasselt JGC, van den Heuvel MM, Schellens JHM, Beijnen JH, Brandsma D, Huitema ADR. Severe cannabinoid intoxication in a patient with non-small-cell lung cancer. Journal of palliative care. 2012;28(1):60-1.
144. Waissengrin B, Urban D, Leshem Y, Garty M, Wolf I. Patterns of use of medical cannabis among Israeli cancer patients: a single institution experience. Journal of pain and symptom management. 2015;49(2):223-30.

145. Walsh D, Kirkova J, Davis MP. The efficacy and tolerability of long-term use of dronabinol in cancer-related anorexia: a case series. Journal of pain and symptom management. 2005;30(6):493-5.
146. Warren PP, Bebin EM, Nabors LB, Szaflarski JP. The use of cannabidiol for seizure management in patients with brain tumor-related epilepsy. Neurocase. 2017;23(5-6):287-91.
147. Webster EM, Yadav GS, Gysler S, McNamara B, Black J, Tymon-Rosario J, et al. Prescribed medical cannabis in women with gynecologic malignancies: A single-institution survey-based study. Gynecologic oncology reports. 2020;34(101652231):100667.
148. Welsh J, Stuart F, Sangster G, Milstead R, Kaye S, Cash H, et al. Oral levonantradol in the control of cancer chemotherapy-induced emesis. Cancer chemotherapy and pharmacology. 1983;11(1):66-7.
149. Zaki P, Blake A, Wolt A, Chan S, Zhang L, Wan A, et al. The use of medical cannabis in cancer patients. Journal of Pain Management. 2017;10(4):353-62.
150. Zhou ES, Nayak MM, Chai PR, Braun IM. Cancer patient's attitudes of using medicinal cannabis for sleep. Journal of psychosocial oncology. 2021((Zhou, Nayak, Chai, Braun) Department of Psychosocial Oncology and Palliative Care, Dana-Farber Cancer Institute, MA, Boston, United States(Zhou) Division of Sleep Medicine, Harvard Medical School, MA, Boston, United States(Chai) Division of Medical Toxic):1-7.
151. Zolotov Y, Eshet L, Morag O. Preliminary assessment of medical cannabis consumption by cancer survivors. Complementary therapies in medicine. 2021;56(9308777, c6k):102592.
152. Zylla DM, Eklund J, Gilmore G, Gavenda A, Guggisberg J, VazquezBenitez G, et al. A randomized trial of medical cannabis in patients with stage IV cancers to assess feasibility, dose requirements, impact on pain and opioid use, safety, and overall patient satisfaction. Supportive care in cancer : official journal of the Multinational Association of Supportive Care in Cancer. 2021;29(12):7471-8.
